# Fast calcium transients in dendritic spines driven by extreme statistics

**DOI:** 10.1371/journal.pbio.2006202

**Published:** 2019-06-04

**Authors:** Kanishka Basnayake, David Mazaud, Alexis Bemelmans, Nathalie Rouach, Eduard Korkotian, David Holcman

**Affiliations:** 1 Computational Biology and Applied Mathematics, Institut de Biologie de l'École Normale Supérieure, Paris, France; 2 Center for Interdisciplinary Research in Biology, Collège de France, Centre National de la Recherche Scientifique UMR 7241, Institut National de la Santé et de la Recherche Médicale U1050, Labex Memolife, Paris Sciences et Lettres Research University, Paris, France; 3 Commissariat à l’Energie Atomique et aux Energies Alternatives, Département de la Recherche Fondamentale, Institut de biologie François Jacob, Molecular Imaging Research Center and Centre National de la Recherche Scientifique UMR9199, Université Paris-Sud, Neurodegenerative Diseases Laboratory, Fontenay-aux-Roses, France; 4 Department of Neurobiology, Weizmann Institute of Science, Rehovot, Israel; 5 Faculty of Biology, Perm State University, Perm, Russia; 6 Department of Applied Mathematics and Theoretical Physics, Churchill College, University of Cambridge, Cambridge, United Kingdom; Columbia University, United States of America

## Abstract

Fast calcium transients (<10 ms) remain difficult to analyse in cellular microdomains, yet they can modulate key cellular events such as trafficking, local ATP production by endoplasmic reticulum-mitochondria complex (ER-mitochondria complex), or spontaneous activity in astrocytes. In dendritic spines receiving synaptic inputs, we show here that in the presence of a spine apparatus (SA), which is an extension of the smooth ER, a calcium-induced calcium release (CICR) is triggered at the base of the spine by the fastest calcium ions arriving at a Ryanodyne receptor (RyR). The mechanism relies on the asymmetric distributions of RyRs and sarco/ER calcium-ATPase (SERCA) pumps that we predict using a computational model and further confirm experimentally in culture and slice hippocampal neurons. The present mechanism for which the statistics of the fastest particles arriving at a small target, followed by an amplification, is likely to be generic in molecular transduction across cellular microcompartments, such as thin neuronal processes, astrocytes, endfeets, or protrusions.

## Introduction

Extreme statistics describes the distribution of rare events, such as the first ions to find a small target [1, 2], which are difficult to detect experimentally in biological microdomains. However, indirect signatures can be derived from their statistical properties. We examine here the role of rare events associated with calcium dynamics in dendritic spines that are local microdomains located on the dendrites of neuronal cells. Spines can form synaptic connections that transmit neural activity [3, 4]. Here, we describe specifically how the fastest calcium ions define the time scale of calcium transduction when it is followed by an amplification step. Regulating this fast event has many consequences in the induction of plastic changes. Indeed, calcium increases are often restricted to the spine head isolated from the dendrite, enabling the induction of local synapse-specific calcium-dependent plasticity leading to α-amino-3-hydroxy-5-methyl-4- isoxazolepropionic acid (AMPA) receptor accumulations [5, 6]. A fast and localized amount of calcium ions is necessary to induce ATP production from mitochondria to supply the energy required to maintain homeostasis [7–9].

Spines are characterized by the diversity of their shapes, sizes, and the presence or absence of different structural components and organelles such as an endoplasmic reticulum (ER). During synaptic plasticity, spine morphology [10–12] can change, leading to an increase/decrease of the head size [13] or an elongation/retraction of its neck. Neck elongation can further lead to electrical and biochemical isolation from their parent dendrites [14–21]. Several spines may contain smooth ER, fragmented in a compartment called the spine apparatus (SA), that regulates calcium ion concentrations by storing or releasing them [22, 23] and modulate synaptic inputs [24, 25]. The SA is monitored by the actin-associated protein synaptopodin (SP) that can modulate calcium kinetics [26–28].

After calcium ions enter dendritic spines, they can bind to endogeneous buffers, get extruded by pumps into the extracellular space, or be pumped into the SA by the sarco/ER calcium-ATPase pumps (SERCA3). Calcium ions can also induce calcium release (denoted calcium-induced calcium release [CICR]) from internal SA stores through the Ryanodine receptors (RyR) [23, 29]. However, the specific calcium regulation by SA remains unclear due to the fast dynamics and the spine nanometer scale organization. We recall that the time scale of calcium diffusion transient during long-term plasticity [30] induction is of the order of hundreds of milliseconds [3, 31] but not faster. However, back-propagating action potential can also elicit fast calcium transients, leading to long-term enhancement of calcium transients in dendritic spines [32].

We show here that the mechanism involved in fast calcium transient (faster than tens of milliseconds) relies on a new mechanism associated with the extreme statistics of the fastest ions that we describe here. For that purpose, we develop a computational model for calcium ion dynamics and use stochastic simulations to interpret uncaging and fluorescent recording. To simulate calcium dynamics in synapses and dendritic spines, there are two possible approaches that have been used previously: deterministic reaction-diffusion equations [33, 34] or stochastic modeling [35–39]. With our approach based on stochastic modeling, we obtain here a new understanding of fast calcium transients: after calcium ions are released inside the spine head using flash photolysis of caged calcium, the concentration increase at the dendrite is faster in spines containing a SA compared to those in which it is absent. This is a paradox, as the SA should obstruct the passage from the spine head to the dendrite and prevent calcium ions from diffusing. To address the paradox, we use stochastic simulations to show that after calcium release in the spine head, under the hypothesis that RyRs are located at the base and SERCA in the head of spine, the fastest ions arriving at the base determine the time scale of calcium transients due to an amplification step. We further confirm this hypothesis experimentally using imaging in culture and slice hippocampal neuron.

Furthermore, we find that the distribution of arrival times of the fastest ions depends on the initial number of calcium ions, which is a signature of extreme statistics and rare events. Finally, we suggest that molecular activation initiated by the fastest particles is a generic mechanism in molecular transduction that can occur in cellular microcompartments, such as protrusions or astrocytic endfeets. This mechanism is likely to define the time scale of biochemical activation in nano- and microdomains, when the source of diffusing particles and the binding targets are spatially separated.

## Materials and methods

### Ethics statement

Animal handling was done in accordance with the guidelines of the Institutional Animal Care and Use Committee (IACUC) of the Weizmann Institute of Science and College de France and the appropriate Israeli and French law. The Weizmann Institute is accredited by AAALAC. The Weizmann Institutional Animal Care and Use Committee approved this study, conducted with cultured hippocampal neurons.

### Animals statement

Experiments were carried out according to the guidelines of the European Community Council Directives of January 1, 2013 (2010/63/EU) and of the local animal welfare committee (certificate A751901, Ministere de l’Agriculture et de l’Alimentation). All efforts were made to minimize the number of animals used and their suffering. Mice (Mus musculus) were group housed on a 12-h light/dark cycle.

### Experimental procedure

#### Calcium uncaging and immunostaining procedure in cultured hippocampal neurons

Cultured hippocampal neurons were transfected with DsRed and loaded with NP-EGTA AM (caged calcium buffer) after which several specific cells were microinjected with Fluo-4 calcium sensor. We expect no more than 50 μM Fluo-4 after sharp pipette unicellular microinjection. Pipette resistance was 30–50 MΩ, the dye concentration in the pipette was 1 mM, and the duration of injection was about 1 s. Calcium imaging was done at 30 °C.

UV laser was directed to either spine heads or the basal dendritic shafts. Following the flashes of ND-YAG UV laser (4 ns, 330 nm) focused into a region of about 0.5 μM in diameter, the released calcium signals could be detected and line scanned using confocal microscope at the rate of 0.7 ms/line. Other sources of calcium fluctuations were blocked using TTX (1 μM), DNQX (10 μM), and APV (20 μM). No special image processing has been applied. Confocal offset function has been used to adjust the background levels for immunostaining. Each recording channel has been adjusted independently, using range indicator. The "optimal signal" imaging mode has been used, in which each separate imaging track is illuminated with its specific wavelength one by one, without mixing the excitation lines.

Following the experiment, cultures were fixed in 4% paraformaldehyde and immunostained for SP (green staining). The same cell regions, containing recorded spines, were identified and imaged. For immunostaining, cover glasses bearing transfected primary hippocampal cells were washed briefly with standard extracellular solution (NaCl 129 mM, KCl 4 mM, MgCl 21 mM, CaCl 22 mM, glucose 10 mM, and HEPES 10 mM). Cultures were then fixed with 4% paraformaldehyde and 4% sucrose in 0.1 MPBS, pH 7.4, for 20 min and washed with PBS thoroughly. Cultures were incubated for 1 h with 10% normal goat serum (NGS) in 0.1% Triton X-100 containing PBS to reduce unspecific staining and subsequently incubated for 24 h at 4 °C in rabbit anti-SP antibody (SE-19, Sigma; 1:1,000, 10% bovine serumalbumin, 0.1% Triton X-100 in PBS) and/or rabbit anti-RYR1 antibody (gift from Dr. Shoshan-Barmatz BGU, Israel, 1:250, 10% goat serum in PBS) (Shoshan-Barmatz and colleagues, 2007). Anti-SERCA2 N-19 and anti-SERCA3 PL/IM430 were from Santa Cruz Biotechnology. Cultures were incubated for 1 h with Alexa 568-labeled or Alexa 633-labeled goat anti-rabbit antibody (Invitrogen; 1:200, 10% bovine serum albumin, 0.1% TritonX-100). Cover slips were washed again, transferred onto glass slides, and mounted for visualization with antifading mounting medium.

For double or triple immunolabeling, cultures were incubated in a mixture of rabbit anti-SP antibody and other antibodies visualized at appropriate wavelengths. After washing, sections were incubated first with anti-rabbit secondary antibody, washed again, and then incubated with anti-mouse secondary antibody. In all cases, secondary and tertiary dendritic segments were visualized. Confocal image stacks were recorded using a Zeiss LSM510 laser-scanning microscope or Zeiss 880 LSM airy scan confocal using a 40 oil-immersion objective lens (1.4 numerical aperture [NA]) and 4-scan zoom. Detector gain and amplifier were initially set to obtain pixel densities within a linear range. Up to 25 images were recorded per stack. All images in this study were sampled at a rate more than two times the ideal Nyquist rate.

Finally, immunostained cells were visualized using two confocal systems: (1) inverted LSM 510 Zeiss, 100x, 1.4 NA, oil-immersion objective, zoom 4–6, z-stacks of 0.7-μM thin optical sections, 4–8x average, 2,000 x 2,000 pixel window, range indicator used for optimized illumination, and 488, 543, 633 laser lines used for excitation and (2) upright, LSM 880 Zeiss, 63x 1.4 NA, oil immersion objective, GaSP PMT, Airyscan, 0.7-μM optical sections, and 405, 488, 543 wavelengths. For the statistical analysis, 18 SP+ and 26 SP− spines were analyzed.

#### Glutamate uncaging

Cultured hippocampal neurons have been transfected with DsRed (1 μg/well) plasmid using Lipofectamine 2000 at the age of 6–7 d in vitro (DIV). Transfected cells displayed no apparent differences in morphology, spine density, and survival compared to GFP transfected cells. Cells were left to grow in the incubator at 37 °C, 5% CO_2_ and were used for experimentation at 14–17 DIV.

Cultures were placed in the imaging chamber, controlled by an automated X-Y stage (Luigs and Neumann). Neurons were imaged on the stage of an upright Zeiss PASCAL confocal microscope using an Olympus 63x water-immersion objective (0.9 NA) and 4x scan zoom. Temperature in the recording chamber was adjusted to 30C. Standard recording medium contained (in mM): 129 NaCl, 4 KCl, 1 MgCl_2_, 2 CaCl_2_, 10 glucose, 10 HEPES, pH adjusted to 7.4 with NaOH, and osmolarity to 310 mOsm with sucrose. K+ Fluo-4 solution (200 μM, Invitrogen) was injected into neurons with sharp micropipettes with resistance of about 5 MOhm during 3 s and allowed to diffuse for 1 h before imaging. Flash photolysis of caged molecules has been described elsewhere in more details [17]. A UV laser (New Wave, air-cooled ND:YAG), emitting 355 nm, 4 ns single light pulses, was focused through the objective lens (63x, 0.9NA Olympus, water-immersion) into a spot of <1 μm^3^. The UV spot is localized using a parallel red laser light (633 nm), directed through the sane optical axis. Single UV pulses could be applied repeatedly without noticeable tissue damage. MNI-caged glutamate, Tocris Bioscience, 0.5 mM was used for the uncaging procedure. The laser was pointed to the area adjacent to a randomly chosen dendritic spine with relatively long (0.8–1 μm) neck length to minimize the uncaged glutamate effect on the shaft due to diffusion toward the parent dendrite. Line scans through the spine head and the dendrite were recorded at the rate of 0.7 ms/line with pinhole adjusted to 1.5 μm thick optical section. No bleaching or photo damage was seen during the recording. Note that we did calibrate the uncaging pulse to obtain physiological synaptic responses, which we recorded with electrophysiology together with the uncaging. Thus, the glutamate uncaging procedure induces currents similar to synaptic events (see also Results).

After the experiment, cover glasses with transfected hippocampal cells were washed briefly with standard extracellular solution. Cultures were then fixed with 4% PFA and 4% sucrose in 0.1 MPBS, pH 7.4, for 20 min, and washed with PBS thoroughly. Cultures were incubated for 1 h with 10% normal goat serum in 0.1% Triton X-100 containing PBS and subsequently incubated for 24 h at 4 °C in rabbit anti-SP antibody (SE-19, Sigma; 1:1,000, 10% BSA in PBS). Then cultures were incubated for 1 h with Alexa-488-labeled goat anti-rabbit secondary antibody (Invitrogen; 1:200). Coverslips were washed again, transferred onto glass slides, and mounted for visualization with antifading mounting medium. The same transfected neurons and the same dendritic spines as imaged during the live experiment were identified and the spines were classified as SP+ or SP−, depending on the presence of immunostaining in the heads and/or necks of the analyzed spines.

#### AAV production and injection

One GFP cassette was placed under the control of a hSynapsin-specific promoter in an AAV shuttle plasmid containing the inverted terminal repeats (ITR) of AAV2. Pseudotype serotype 9 AAV particles were produced by transient cotransfection of HEK-293T cells. Viral titers were determined by quantitative PCR amplification of the ITR on DNase-resistant particles and expressed as vector genomes per mL (vg/mL).

Two-month old C57Bl6 mice were anesthetized with a mixture of ketamine (95 mg/kg; Merial) and xylazine (10 mg/kg; Bayer) in 0.9% NaCl and placed on a stereotaxic frame under body temperature monitoring. AAVs were diluted in PBS at a concentration of AAV-hSynapsin-GFP 1.02 × 10^13^vg/ml, and 1 μl of virus was stereotaxically injected unilaterally into the hippocampal region at a rate of 0.2 μl/min, using a 29-gauge blunt-tip needle linked to a 2-μl Hamilton syringe (Phymep). The stereotaxic coordinates to Bregma were antero-posterior: +2 mm; lateral: −1.5 mm; and dorso-ventral: −2 mm. At the end of the injection, the needle was left in place for 5 min before being slowly removed. The skin was sutured, and mice recovery was checked for the next 24 h. After 2 weeks, the mice were sacrificed, and the brains were extracted after 2% paraformaldehyde/PBS intracardiac perfusion.

#### Immunoshistochemistry

40-μm thick brain slices were cut with a Leica microtome. Brain slices were permeabilized and blocked for 2 h in 0.25% Triton/0.2% Gelatine in PBS (blocking solution) at room temperature. Primary and secondary antibodies were diluted in blocking solution and incubated overnight at 4 °C and mounted in Fluoromount-G mounting medium. The following primary antibodies were used (anti-SP [Rabbit, 1:100, Sigma-Aldrich SE-19], anti-Ryanodin Receptor [Mouse, 1:100, Abcam ab2827], anti-Serca3 [Mouse, 1:100, Sigma-Aldrich WH0000489M1], anti-GFP [chicken, 1:600, Aves 1020]) in combination with the following secondary antibodies (anti-chicken 488 [Goat, 1:300, Invitrogen, A11039], anti-rabbit 555 [Goat, 1:300, Invitrogen, A21439], anti-mouse 647 [Goat, 1:300, Invitrogen, A21235]). Z-stacks images were taken using a super-resolution STED microscope (Abberior Instruments GmbH) and analyzed using ImageJ software. Identification and localization of punctae was done when the brightest punctae staining in the z-axis was within the dendritic spine to limit the analysis of punctae located below or above the spine.

#### Super-resolution STED imaging in brain slices

STED imaging was performed using a custom upright STED microscope (Abberior Instruments). The microscope is based upon a Scientifica microscope body (Slice Scope, Scientifica) with an Olympus 100x/1.4NA ULSAPO objective lens. It comprises a scanner design featuring four mirrors (Quad Scanner, Abberior Instruments). 488 nm, 561 nm, and 640 nm excitation lasers are available (Abberior Instruments, pulsed at 40/80 Mhz). Two STED-lasers at 595 nm (MPB-C, cw), and 775 nm (MPB-C, pulsed at 40/80 MHz) are at disposal. The conventional laser excitation and STED laser beams are superimposed using a beam-splitter (HC BS R785 lambda/10 PV flat, AHF Analysetechnik).

Common excitation power with pulsed excitation ranges from 10–20 uW with STED power intensities of up to 200 mW in the focal plane. Gated STED is possible where STED at 595 nm is always gated STED due to the cw-laser (typical gate delay of 1 ns to 2 ns, depending on desired signal level and resolution).

#### Statistics

All data are expressed as mean ± SEM. Statistical significance for within-group comparisons was determined by one-way ANOVAs (followed by Tukey’s post-test), whereas unpaired *t* tests were used for between-group comparisons.

### Stochastic simulations of calcium ions in a dendritic spine

We model a dendritic spine with a spherical head connected to a narrow, cylindrical neck, as described in [17, 37, 40]. The SA present in SP+ spines are modeled also with a similar geometry with a neck and a head positioned inside the spine. Calcium ions are described as Brownian particles following the Smoluchowski limit of the Langevin equation $\dot{X}=\sqrt{2D}\dot{w}$, where *W* is the Wiener white noise. This motion of particles in the spine is simulated using the Euler’s scheme: $x_{n}=x_{n-1}+\sqrt{2D\Delta t}\cdot \eta$, in which *X*_*t*_ = {*X*, *Y*, *Z*} is the position of a particle at time *t*, while *η* is a three-dimensional normal random variable. *D* is the diffusion coefficient of calcium ions in the cytoplasm, and Δ*t* is the time step. (All parameter values are summarized in S1 Table). We neglected here the baseline of free calcium concentration, and we released *N* = 1,000 particles (leading to an initial calcium concentration of 0.4 μM in the spine head). Ions can diffuse inside the cytoplasm, and they are reflected at the surfaces of the spine and the SA (Snell–Descartes reflection). To replicate the uncaging experiments, we use either the center of the ball or the base of the cylindrical neck as the initial positions of particles. Ions arriving at the dendritic shaft located at the base are considered to be lost and do not return to the spine during the time scale of the simulations (absorbing boundary). The inner surface of the spine head contains absorbing circular disks with a 10-nm radius, which models calcium pumps. In all simulations, we kept the number of pumps in the head fixed at the value 50, as calibrated from a pure diffusion model (see S1 Fig). The number of trials for each simulation is shown in S2 Table. Our code is written in Python 2.7 and is available at http://bionewmetrics.org/simulations-of-spine-calcium-transients-with-er/.

#### RyRs

RyRs are activated upon the arrival of the first two ions to a small absorbing disk of size *a*_*RyR*_ = 10 *nm* (see S2 Fig). We positioned *n*_*R*_ = 36 receptors for the simulations organized in four rings in the SA neck, each containing six receptors. The other 12 are located on the SA component parallel to the dendritic shaft. After a receptor opens, it releases instantaneously a fixed number of calcium ions *n*_*Ca*_, which are positioned at the center of the receptors. Following this release, a RyR enters into a refractory period that lasts 3 to 6 ms, during which it is modeled as a reflective boundary for free diffusing ions. After calibration, we find that calcium ions should be released with a delay of 0.25 ms after the arrival of a second ion to the RyR-binding site.

#### SERCA pumps

We model SERCA pumps as absorbing disks of size ***a***_***SERCA***_ = **10 *nm***. When a calcium ion arrives to the disk, it is bound indefinitely. If a second ion arrives, both are absorbed immediately, and the transporter is frozen in an inactive state (see S3 Fig). We positioned 36 SERCA pumps uniformly distributed on the upper hemisphere of the spine head.

#### Mean first passage time of ions to the base of a spine

For a Brownian particle released in the spine head, the mean arrival time to the base of a spine has been computed asymptotically [39]

$$\underline{\tau }=\frac{V}{4Da}[1+\frac{a}{\pi R}log(\frac{R}{a})]+\frac{L^{2}}{2D}+\frac{VL}{\pi Da^{2}},$$ (1)

in which *D* is the diffusion coefficient, *V*, *a*, *R*, and *L* are the volume of the head, spine neck radius, head radius, and the total length of the neck, respectively. We refer to S1 Table for the parameter values, from which we estimated $\underline{\tau }\approx 120\;ms$.

## Results

### Fast calcium transient in spines with and without a SA are not due to classical diffusion

**Fig 1:**
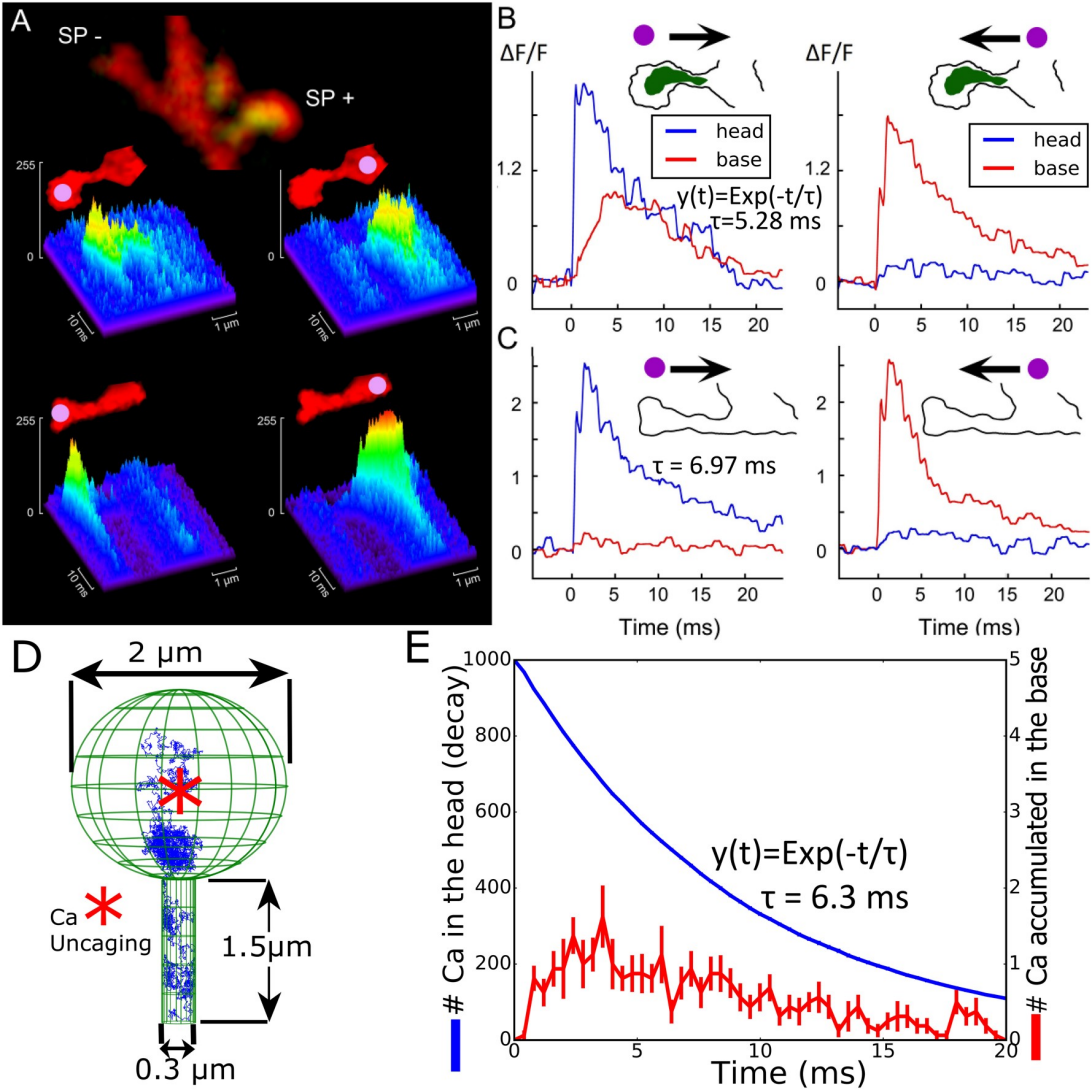
Calcium transient in dendritic spines with and without a SA. (A) Examples of line scans in two neighboring spines of about same length, following flash photolysis of NP-EGTA in spine heads (left) and the parent dendrite (right). At the end of the experiment, the cultures were immunostained for SP (green staining). The same cell regions, containing recorded spines, were identified and imaged. Some spines (about 25%) of total mushroom spines contained SP puncta (SP+) while in the others (SP−), a clear puncta could not be seen. (B and C) Individual traces of calcium transients for the same spine heads (blue) and the parent dendrites (red) are shown on panel A. Schematic contours of two spines, containing (SP+) and not containing (SP−) puncta are shown on the top of the graphs. The arrows indicate the possible direction of calcium diffusion from the focus of uncaging (purple dots). There is a clear signal transfer from the spine head to the parent dendrite in the SP+ spine and such signaling is absent if the focus of uncaging is set in the dendrite (B). For the SP− spine, calcium signaling in both directions looks the same (C). The decay times *exp*(−*t*/*τ*) for calcium in the head were fitted for 19 (for SP+) and 27 (for SP−) experiments. (D) Stochastic simulations of calcium ions in a dendritic spine without a SA; initial position of calcium ions (red star) and a trajectory (blue) are shown. The surface of the head contains 50 absorbing circular calcium pumps with a 10-nm radius (not shown). (E) Simulated calcium transient following the model in (D). Calcium ions propagate from the head to the neck. NP-EGTA, *o*-Nitrophenyl egtazic acid; SA, spine apparatus; SP, synaptopodin.

**Fig 2:**
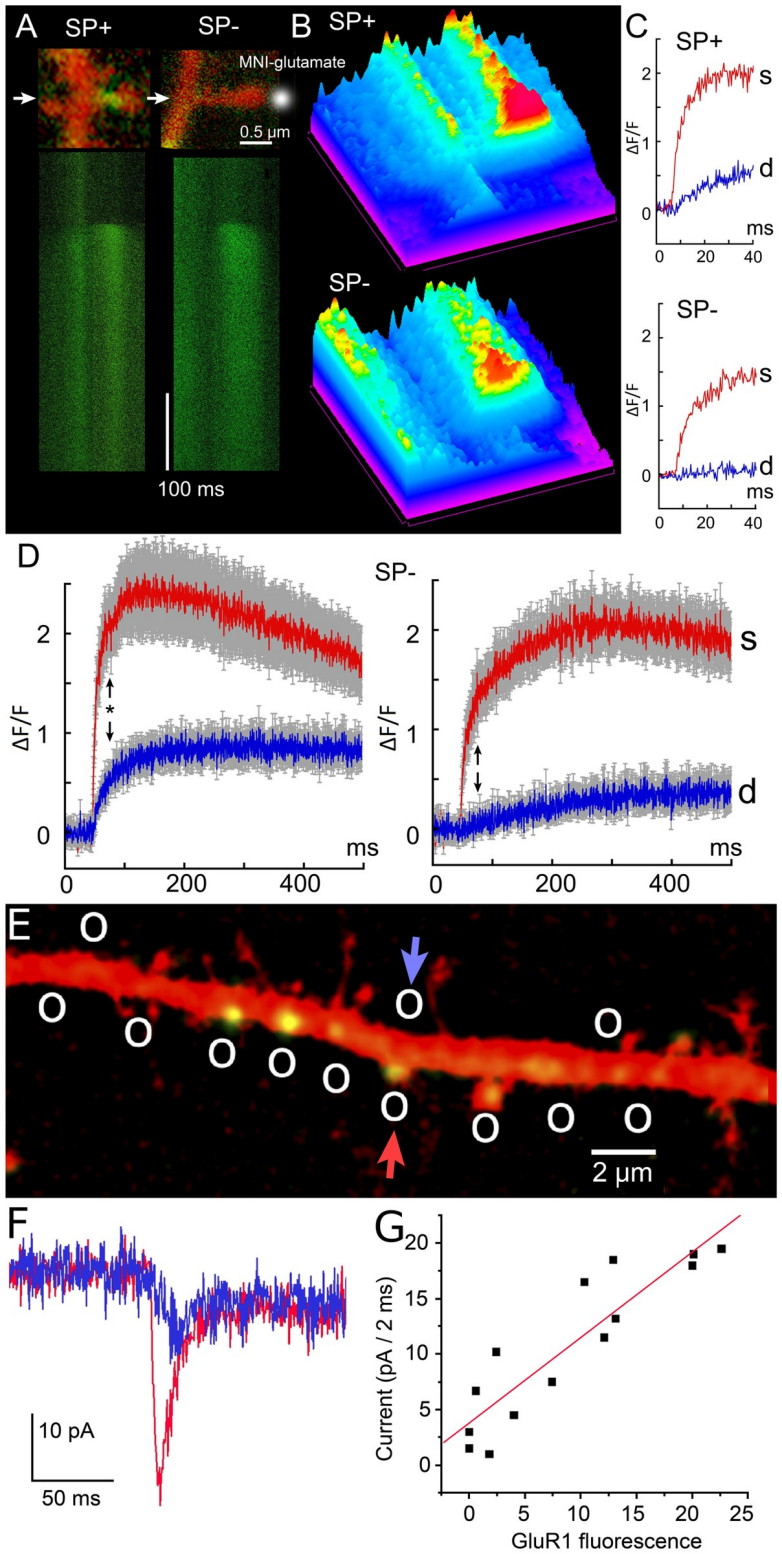
Effect of MNI-caged glutamate photolysis on spine-dendrite coupling in SP positive and negative spines. (A top) Examples of two SP+ and SP− spines as revealed by immunocytochemical analysis, done after the recording experiments. Transfected cultured hippocampal cells were microinjected with 200 μM K+ Fluo-4 and calcium transients as fast 0.35 ms/line line scans (A bottom) were recorded following single 4 ns 355 nm Nd:YAG laser flash (third harmonic of 1,064 nm). (B) Same line scans, presented as spectrum-colored surface plots, oriented from bottom right to top left with magenta–blue corresponding to low fluorescence and yellow–red to high Fluo-4 fluorescence. (C) Examples of the initial stage of calcium transients (during the first 30 ms) from SP+ and SP− spines. (D) Summarized graphs presenting 23 SP+ spine/dendrite pairs and 22 SP− spine/dendrite pairs. Averaged spine lengths were 1.1 ± 0.13 μm for SP+ and 1.15 ± 0.15 μm for SP− spines. Note faster and higher calcium rise in the dendrites SP+ versus SP− parent dendrites and faster decay time in the SP+ spine heads. Black arrows indicate 80 ms: *P* value based on ANOVA for (SP+/SP−) spines is 0.011, while for (SP+/SP−) dendrites is 0.038, respectively. (E) Multiple uncaging locations (circles) of glutamate along the dendrite (transfected with GluR-1 [AMPA receptor] marked in green, to visualize the accumulation of GluR1 and transfected with DsRed for visualizing dendrite morphology), from typical cultured pyramidal neurons. Glutamate is uncaged at several GluR-positive and GluR-negative locations. (F) Patch clamp responses to glutamate uncaging located by the arrows (in panel E) at a dendritic spine (red, GluR-1-positive) and on the dendritic shaft (blue, GluR-1-negative). (G) Overall correlation between GluR-1 fluorescence and recorded current following uncaging [41]. Data used for generating D and G is available in S1 Data. AMPA, α-amino-3-hydroxy-5-methyl-4- isoxazolepropionic acid; GluR-1, glutamate receptor 1; SP, synaptopodin.

To investigate the role of the SA, we first released calcium following the flashes of ND-YAG UV laser to uncage calcium in dendritic spines from hippocampal neurons (Fig 1A). After the experiment, the cultures were fixed using 4% paraformaldehyde and immunostained for SP to identify spines containing SA (see Materials and methods). About 25% of total mushroom spines contained SP puncta (SP+) while in the others (SP−) clear puncta could not be seen. Note that medium spines (of about 1–1.5 μm in length) are studied. The transient fluorescence signal reveals the influence of the SA on calcium dynamics as shown in Fig 1B and 1C (see also S4 Fig). The calcium decay time in the head is well approximated by a single exponential [39] with a time constant *τ* = 5.28 *ms* in SP+ compared to *τ* = 6.97 ms for SP−, showing that the SA does not influence the extrusion rate from the spine head, probably because its obstruction is not completely occluding the passage from the head through the head-neck junction. However, the elevation of calcium at the base was much different, leading to high and very fast elevation in the case of SP+, a phenomena that is in the focus of the present study. Finally, uncaging at the base of the spine leads to the same response in the head for SP+ and SP−, suggesting that the privileged calcium response occurs only in the head–neck direction when a SA is present. The asymmetry found here between releasing either in the spine head or at in the dendrite is specific to calcium, as shown in S4 Fig. We confirm that similar calcium transients can be induced by caged glutamate only in SP+ dendritic spines, as classified by the presence of immunostaining in the heads and/or necks, as shown in Fig 1A–1D. Note that the glutamate evoked responses are similar to ones induced by a synaptic event Fig 2E–2G. Thus, we conclude that fast calcium transients at the base of the SP+ spines did not depend on the mode of induction, as they could be induced by calcium and glutamate uncaging with similar time scale.

For analyzing the calcium transient further, we used stochastic simulations, for which ions are treated as Brownian particles (see Materials and methods) and released at the center of the spine head (Fig 1D and 1E). Using a single exponential approximation, we obtained a decay time in the head *τ* = 6.3 *ms*, which is comparable to the one obtained experimentally (see S1 Fig for the calibration of calcium pumps in the absence of RyRs and SERCAs). To conclude, fast calcium transient of less than 20 ms in spines with no SP is well reproduced by stochastic simulations, but the calcium increase at the dendrite base for spines with an SA is much faster than the mean arrival time of calcium ions. This effect is surprising because it occurs despite the serious SA obstruction that should prevent calcium ions from passing easily through the neck. We shall now investigate the mechanism for this fast increase.

### The fast calcium transient is generated by CICR and the asymmetric distribution of RyR on the SA

**Fig 3:**
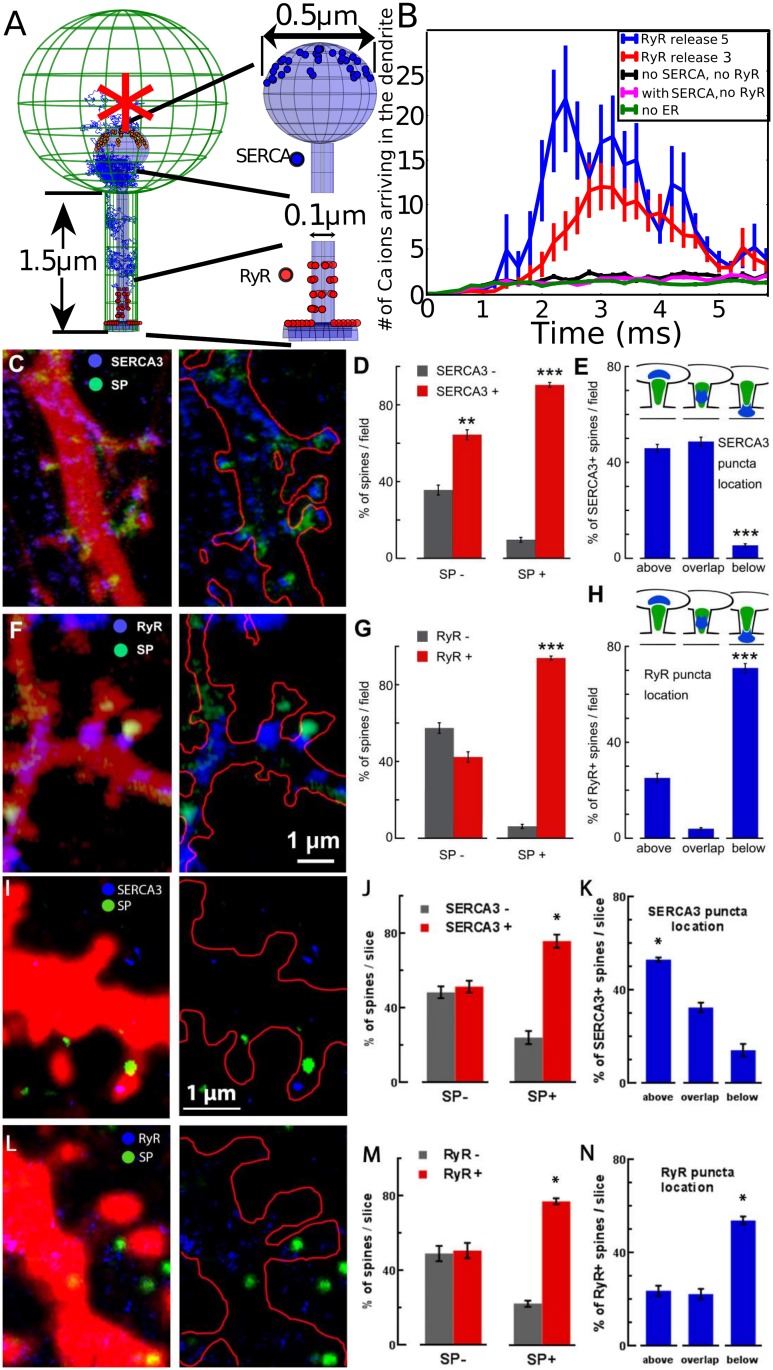
Modeling and simulation of Calcium dynamics to discover the distribution of RyR and SERCA in the SA. (A–B) Stochastic simulations of calcium ions; (G) model of a spine containing an SA with SERCA pumps and RyRs (right: magnifications show the distribution of 36 SERCA pumps in the upper hemisphere of the SA head and 36 RyRs located on the SA at the base, 12 on the shaft, and 24 in the neck, organized in four rings containing six randomly distributed receptors). Ions are initially released at the center of the head (red star). (B) Calcium ions arriving at the base of the dendrite following release in the head and release from RyRs (three and five ions per activated receptors). (C–H) Distributions of SERCA3 and RyRs in dendritic spines depend on the presence of SP puncta. (A) and (F) Cultured rat hippocampal neurons were cotransfected with DsRed (red color on the right panels and red contour on the left panel) and SP (green puncta) and immunstained against SERCA3 and RyR. In SP-occupied dendritic spines, top head locations of SERCA3 (blue staining in [D]) and basal locations of RyR (blue staining in [F]) are confirmed. Gray columns versus red columns compare the presence of SERCA3 immunostaining (D) and RyR immunostaining (G) in SP− (left bars) and SP+ (right bars) spines. Both the number of SERCA3− versus SERCA3+ as well as RyR− versus RyR+ spines is given in percent per standard field (*N* = 41 fields for [D] and 67 fields for [G]). Higher percentage of SERCA3+ and RyR+ spines in the SP+ groups (*t* probability < 0.01 for [D] and < 0.001 for [G]). (E) Specific location of SERCA3 in SP+ dendritic spines. Number of spines is given as percentages of total SP+ and SERCA3+ spines per standard field, which is taken as 100%. Fields are the same as in (D). Schematic representations of SERCA3 and SP puncta "typical" locations are shown on the top of the panel (E). Prevalence of SERCA3 puncta location above the SP puncta or overlapping with it (left and middle bars). (H) Location of RyR in SP+ dendritic spines. Again, the number of spines is given as percentages when the total SP+ and RyR+ spines per standard field is taken as 100%. Fields are the same as in (G). Schematic representations are shown on the top of the panel (H). Prevalence of RyR puncta located below the SP puncta (right bar). (I–N) Distributions of SERCA3 and RyR puncta in dendritic spines of hippocampal neurons from adult mouse brain slices also depend on the presence of SP puncta. (I) and (L) C57Bl6 mouse hippocampal neurons infected with AAV-hSynapsin-GFP virus (red color on the left panels and red contour on the right panels) and immunostained against SERCA3 or RyR and SP. Representative images show the top head locations of SERCA3 (blue staining in [I]) and basal locations of RyR (blue staining in [L]) in SP occupied dendritic spines (green staining in both [I] and [L]). (J) and (M) Gray versus red columns compare the presence of SERCA3 or RyR immunostainings in SP− (left bars) and SP+ (right bars) spines. Both numbers of SERCA3- versus SERCA3+ and RyR− versus RyR+ spines are given in percent of spines per brain slice (between 7 to 17 fields are analyzed for each sample for a total of 548 and 376 spines analyzed for SERCA3 and RyR conditions, respectively). There is significantly higher percentage of SERCA3+ and RyR+ spines in the SP+ groups (*P* < 0.05 for both conditions). (K) and (N) There is a significant prevalence of SERCA3 puncta location above SP puncta and RyR puncta location below SP puncta (one-way ANOVA test with multiple comparison, *P* < 0.05). The number of spines is given as percentages of the total SP+ and SERCA3/RyR+ spines. The fields of (K) and (N) are the same as in (J) and (M), respectively. Data of the panels D, E, G, H, J, K, M, and N are available in S1 Data. AAV, Adeno-associated virus; GFP, green fluorescent protein; RyR, Ryanodyne receptor; SA, spine apparatus; SERCA, sarco/ER calcium-ATPase; SP, synaptopodin.

To gain intuition and clarify how the SA could affect the calcium transient, we first run stochastic simulations similar to the ones we used in Fig 2F. Ions are released initially in the spine head (red star, Fig 3A). But now we introduce a SA type compartment, where we added 36 SERCA pumps (blue) located on the surface of the SA (head) and 36 RyRs located on the SA at the base (red). While SERCA pumps can uptake calcium ions from the cytoplasm to the ER, RyRs generate a calcium flux from the SA to the cytoplasm when two calcium ions are bound (Materials and methods, S2 and S3 Figs, and S1 Table). Interestingly, and in contrast to the results of Fig 2E, after 1,000 ions are released in the spine head, a significant calcium increase can be observed at the base of the spine in less than 2 ms. This effect is already present when three calcium ions per RyR are released (Fig 3B), and further amplified with five ions, compared to spines with no SA (green curve). Interestingly, this calcium amplification does not depend on the distances between RyRs within the range below 150 nm (see S5 Fig).

At this stage, we proposed to test the prediction of the model and decided to study the distributions of SERCA3 and RyRs located on dendritic spines containing a SP puncta revealed by immunostaining (Fig 3C–3N). We found that SERCA pumps are present in SP+ spines and are located predominantly above the SA inside the spine head (Fig 3C–3E). This is in contrast with the distribution of the RyRs, present in the SP+ and mostly located below the SA at the base of the spine neck (Fig 3F–3H).

In order to confirm these results obtained in vitro, we also analyzed the distributions of SERCA3, RyR, and SP in dendritic spines of adult mouse hippocampi. We performed STED super-resolution imaging in immunostained brain sections from mice injected unilaterally in the hippocampus with the Adeno-associated virus human synapsin green fluorescent protein (AAV-hSyn-GFP). We confirmed that SERCA pumps and RyRs are enriched in SP+ spines, similar to the observations made in neuronal culture: SERCA3 puncta are mostly positioned above SP (Fig 3I–3K), while RyR puncta are preferentially located below SP puncta (Fig 3L–3N).

We thus conclude that fast calcium increase at the base of the spine is due to the calcium release from the SA. This release is induced by the opening of RyRs and triggered by the fastest calcium ions traveling from the head to the neck inside the cytoplasm. Finally, this amplification is possible only when RyRs are mostly located at the base of the spine head and the SERCA pumps in the head. (See below for the confirmation, when calcium ions are released in the dendrite instead of the head).

### Extreme statistic for the fastest ions as a mechanism for activating Ryanodine receptors during calcium transients

**Fig 4:**
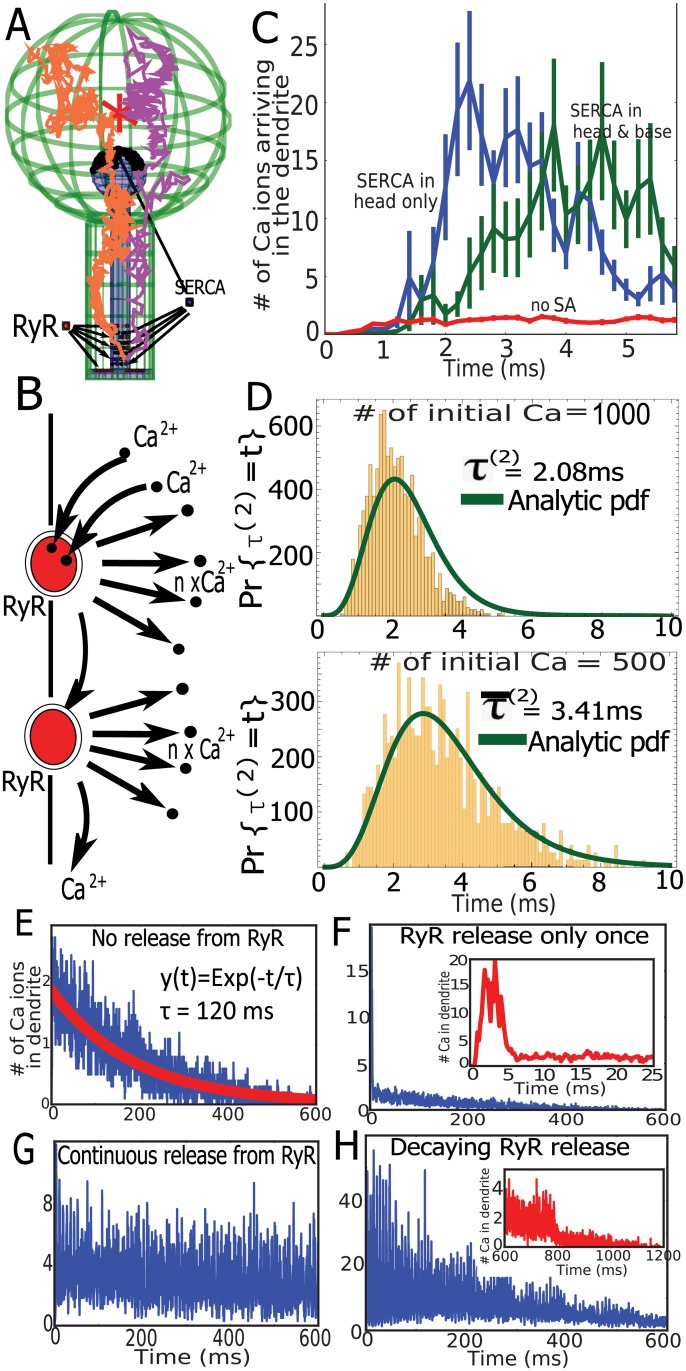
Mechanism of fast calcium release triggered by RyRs. (A) Representation of calcium trajectories in a spine with ER, in which SERCA (blue) are in the head and at the base. Arrangement in four separated layers containing nine of them. RyRs arrangement is described in Fig 3H. (B) Schematic release of calcium ions from RyR opening, triggered by two calcium ions. (C) Dynamics of calcium accumulation in the dendrite in the presence (green) or absence (blue) of SERCA pumps at the base versus dynamics in a spine with no SA (red). (D) Arrival times of the two fastest calcium ions that open the first RyR when the initial numbers are *N* = 1,000 and *N* = 500, superimposed with the analytical solution (not a fit) of Eq 5 (see S1 Text). (E) Transient calcium ions arriving at the base of the dendrite when no RyRs are present but with SERCA and calcium pumps. (F) Similar to (G), all RyRs open and release calcium ions only once and then are deactivated (magnified in the inset). (G) Continuous calcium release following two bound ions at RyR (with a refractory period of 6 ms). (H) Same dynamics as in (G), but the number of calcium released from RyRs is reduced exponentially with time; when RyRs open, *n*_*Ca*_ ions are released and the receptor is desensitized for 6 ms. Initially, *n*_*Ca*_ = 8 and decreases exponentially with a time constant of 360 ms. ER, endoplasmic reticulum; RyR, Ryanodyne receptor; SA, spine apparatus; SERCA, sarco/ER calcium-ATPase.

To clarify the origin of the fast calcium transient observed at the base of a spine, we studied using modeling and simulations the dynamics of RyR opening when ions are released inside the head (Fig 4A). In this stochastic model, a RyR opens when two ions are bound (Fig 4B; see also Materials and methods) and releases calcium ions from SA to the cytoplasm (Fig 4B). Stochastic simulations reveal that in the presence of RyRs, the calcium released in the spine head induces a calcium increase at the base within the first 5 ms (Fig 4C). This effect is modulated by the distribution of SERCA pumps but was clearly due to the presence of RyRs. This result confirms the role of RyRs in generating the fast calcium transient at the base of a spine.

To assess the time scale of RyR activation, we constructed histograms of the first time *τ*^(2)^ to activate RyRs by the binding of two consecutive calcium ions. The distribution of *τ*^(2)^ is shown in Fig 4D. Interestingly, this distribution depends on the initial number of calcium ions. We computed numerically from the histogram the mean time for the first RyR by the first calcium ions, which is 3.4 ms and 2.1 ms, respectively, when 1,000 and 500 ions are initially placed in the head. These times are much faster than the mean time for an ion to arrive at the base of a spine, which is of the order of 120 ms (see Eq 1) or the peak distribution of the arrival for a single ion (around 15 ms). To conclude, we can now understand that the fast calcium transient at the base of a spine containing an SA can be generated by the fast ion arrival, located in the tail of statistical distribution, which therefore selects the fastest among many Brownian particles.

### General theory of extreme statistics for Brownian calcium ions in a cellular microdomain

To further validate the results of stochastic simulations described in the previous section, we computed from the distribution of arrival time for *N* independent Brownian trajectories (ions) at a small binding site inside a bounded domain Ω. This time is defined by *τ*^1^ = *min*(*t*_1_, …, *t*_*N*_), in which *t*_*i*_ are the arrival times of the *N* ions. The arrival probability can be computed when the boundary ∂Ω contains *N*_*R*_ binding sites ∂Ω_*i*_ ⊂ ∂Ω so that the total absorbing boundary is $\partial \Omega_{a}=\overset{N_{R}}{\underset{i=1}{\cup }}\partial \Omega_{i},$ and the reflecting part is ∂Ω_*r*_ = ∂Ω − ∂Ω_*a*_. The probability density function of a Brownian motion is the solution of

$$\begin{array}{l} \frac{\partial p(x,t)}{\partial t}=D\Delta p(x,t)\;for\;x\in \Omega ,t>0\\ p(x,0)=p_{0}(x)\;for\;\;\in \Omega \\ \frac{\partial p(x,t)}{\partial n\;}=0\;for\;x\;\in \partial \Omega_{r}\\ p(x,t)=0\;for\;\;x\in \partial \Omega_{a}. \end{array}$$ (2)

The survival probability, which is the probability that an ion is still not absorbed at time *t*, is given by

$$\text{Pr}\{t_{1}>t\}=\int_{\Omega }p(x,t)\;dx\;,$$ (3)

so that $\text{Pr}\{\tau^{1}=t\}=\frac{d}{dt}\text{Pr}\{\tau^{1}<t\}=N{\left(\text{Pr}\{t_{1}>t\}\right)}^{N-1}\text{Pr}\{t_{1}=t\}$, in which $\text{Pr}\{t_{1}=t\}=\oint_{\partial \Omega_{a}}\frac{\partial p(x,t)}{\partial n\;}\;dS_{\;}$ and $\text{Pr}\{t_{1}=t\}=N_{R}\oint_{\partial \Omega_{1}}\frac{\partial p(x,t)}{\partial n}\;dS_{\;}$. Putting all of the above formulas together, we obtain that the distribution for the first particle to arrive is

$$\text{Pr}\{\tau^{1}=t\}=NN_{R}{\left[\int_{\Omega }p(x,t)dx\right]}^{N-1}\oint_{\partial \Omega_{1}}\frac{\partial p(x,t)}{\partial n\;}dS_{\;},$$ (4)

and the arrival time *τ*^(2)^ for the second ion, which modeled the activation of a RyR, is that of the minimum of the shortest arrival time in the ensemble of *N* − 1 trajectories after the first one has arrived and is given by

$$\text{Pr}\{\tau^{\left(2\right)}=t\}=\int_{0}^{t}\text{Pr}\{\tau^{\left(2\right)}=t\;|\;\tau^{1}=s\}{\left(\int_{0}^{L}\text{Pr}\{\;{\;}_{1}(s)=x_{1}\}dx_{1}\right)}^{N}\text{Pr}\{\tau^{1}=s\}ds.$$ (5)

We plotted in Fig 4D the solution of Eq 5 in which the distribution of arrival time for the first ion Pr{*τ*^1^ = *s*} also accounts for the return of the ion located in the neck, back to the head (see S1 Text for the complete mathematical derivation). We find that the solution superimposes with the stochastic simulations, confirming the consistency of the stochastic simulations and the theory of the extreme statistics. To conclude, this analytical approach further confirms the role of the fastest ions in setting the time scale of CICR by RyR activation. We observe that the typical shortest path is very close to the shortest geodesic, going from the initial position to the RyRs, which is much different compared to the paths associated with the mean arrival time.

### Long-term dynamics of CICR

To further confirm the role of the fastest ions in triggering calcium release, we generated much longer simulations over 600 ms (Fig 4E–4H). In the absence of an SA, we simulated the flux of ions arriving at the dendrite, showing an exponential decay of *τ = 120 ms* (Fig 4E), indeed, in agreement with Eq 1. We note that here, there are no extrusion mechanisms, such as calcium pumps. To evaluate the impact of the SA, we simulated in Fig 4F a single-release event of calcium ions, following RyR activation (five ions are released per receptor). This release is local and affects only the global decay during the first few milliseconds (insets).

When RyRs are releasing a minimum number of two calcium ions with a refractory period of 6 ms, after a fast transient regime, the ensemble of RyR self-entertains (Fig 4G). Indeed, when the SA contains a sufficiently large amount of calcium, the locally released calcium binds to RyRs that open, but the ions disappearing at the base of the spine are not sufficient to prevent this positive feedback loop between calcium and RyRs.

Finally, to account for a local SA calcium depletion, we simulated a decrease in calcium release from the SA, starting from eight ions per RyR. The decay followed an exponential with a decay time of 360 *ms*. After 600 *ms*, the transient regime was completely abolished (Fig 4H).

To conclude, the two fastest ions arriving at a single RyR trigger the release of calcium from SA that induces a local calcium release. The time scale of activation depends on the initial number of released calcium ions in the head, which is the signature of an extreme statistics mechanism. This avalanche mechanism is responsible for the fast and large calcium increase at the base of the spine, when ions are diffusing from the head. Thus, a release of local calcium ions from RyRs amplifies the calcium signal. Furthermore, the calcium transient termination can be attributed to the local SA depletion over a few hundred milliseconds (see S7 Fig).

### Asymmetric calcium dynamics between spine and dendrite

**Fig 5:**
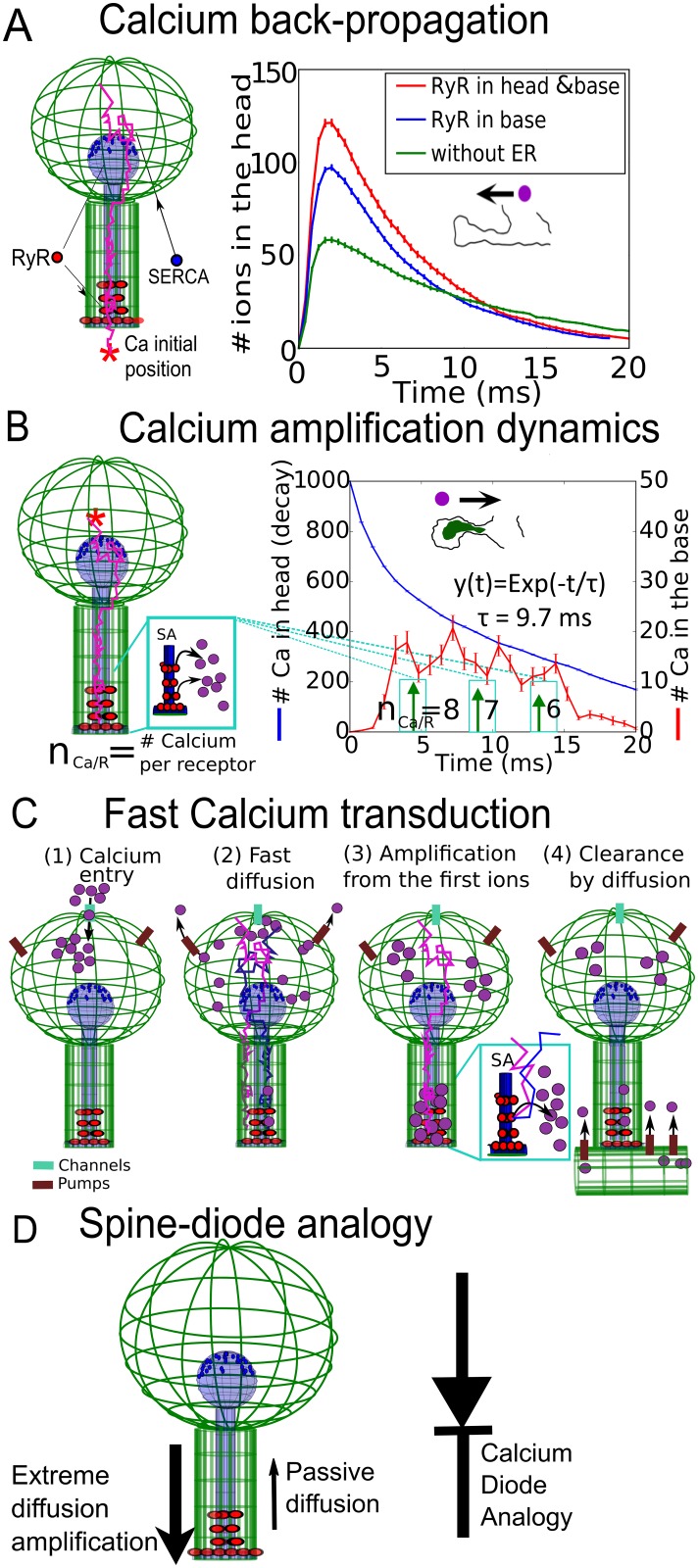
Summary of findings on Calcium transduction in the spine. (A) Left: schematic representation of spine with SERCA pumps in the head and 36 RyRs placed in the head (18) and the neck (18). Calcium ions are released in the dendrite (red star). Right: simulation of calcium transient in the head in the absence of an SA (green), when RyRs are present in both neck and head (red) and only in the neck (blue). (B) Stochastic simulations of calcium transient when taking into account SA depletion: RyRs are releasing with a delay of 0.25 ms, initially eight, seven, and, finally, six ions with an averaged time indicated by the green arrows. The RyR refractory period is 3 ms (see also S6 Fig). (C) Summary of the calcium-diffusion amplification in a dendritic spine with an SA. (D) Diode representation of a spine with an SA. ER, endoplasmic reticulum; RyR, Ryanodyne receptor; SA, spine apparatus; SERCA, sarco/ER calcium-ATPase.

To investigate the consequences of RyR distribution on calcium transients, we replicated the experimental protocol described in Fig 1B–1C with numerical simulations. We ran simulations using the numerical scheme described in Fig 1D, with SERCA pumps located on head of the SA, while calcium ions are released at the base of the spine (red star in Fig 5A). We tested two RyR distributions: (1) RyRs are only at the base of the neck, as suggested from Fig 3F, and (2) RyRs are located in the spine head.

We find that adding RyRs only at the base already increases significantly the calcium transient in the spine head (blue versus green, Fig 5A). If in addition RyRs are added in the head, the calcium transient in the head is further increased (red versus blue). However, when calcium ions are released at the base and measured in the head, calcium transient in the head is not amplified, as showed by our experimental findings (Fig 1B and 1C). Therefore, these stochastic simulations agree with the immunostaining results of Fig 3F, suggesting that there should be no RyRs in the spine head. We note, however, that there is a small difference between the result of our simulations (small increase in the calcium in the head) and the calcium uncaging experiments inside the dendrite, which show in the absence and the presence of an SA only a slight increase of calcium in the head. This difference suggests that removal mechanisms, such as calcium pumps, could be located at the base of the spine, leading to a removal of the fraction of the calcium ions entering the spine versus the one flowing directly. To conclude, the asymmetric distributions of the RyRs contribute to the asymmetry of calcium transmission.

At this stage, we could not access the calcium dynamics inside the ER. However, it could have a drastic consequence on the calcium transient, as shown in Fig 4E–4H (see also S7 Fig, in which we varied the RyR cluster location and restricted calcium concentration in the ER). We thus decided to use the calcium transient signal (Fig 1B, red curve) to recover the local SA depletion at a time scale of 20 ms following calcium release. We had to slightly modify the values of the parameters as now described: we released consecutively eight, seven, and then six ions per RyR with a refractory period of 3 ms between each release (see also S6 Fig). In that case, we could recover the transient kinetics observed experimentally (Fig 5B, red). We remark that the decay from eight, seven, to six ions accounts for the limited amount of available calcium in the ER, which is slowly depleting following consecutive release. The calcium ions were released with a delay of 0.25 ms after the arrival of a second ion to the RyR-binding site. This delay was introduced to account for the reduction of the increase during CICR. We calibrated this delay to account for the experimental time scale. Finally, the refractory period of 3 ms after each release was estimated based on the experimental CICR transient (Fig 1B, left). To conclude, each one of the RyR parameters was optimized using the physiological condition of the calcium transient. This adjustment of parameters reveals a small calcium depletion inside the SA during calcium transient before the SA is depleted in calcium ions. This effect should be considered as a possible prediction of the model.

We also found here that calcium release from the RyRs is delayed by 0.25 ms following the binding of two calcium ions. These results give us an indication of the SA depletion time scale, which is probably at a few tens of milliseconds. Putting the present results together, we describe a novel diffusion–amplification calcium transduction in spines containing an SA (see Fig 5C) and that the SA plays the role of a diode, amplifying ion transmission from the head to the dendrite but not in the opposite direction (Fig 5C).

## Discussion

In the present study, we investigated how SA influences calcium transient inside dendritic spines. We found that calcium increases at the dendrite following uncaging in the head occurs in a few-millisecond time scale. As shown here, this property can be explained by the statistics of the fastest calcium ions, without the need of considering electrodiffusion [19, 42, 43]. The fastest arriving ions open RyRs located at the base of the spine neck, which generate an avalanche through a CICR from SA. This local avalanche leads to an accelerated amplification of the calcium signal before the remaining ions diffuse from the head. Previously, RyRs were shown to contribute to calcium transients in the spine’s cytosol [32] in the context of back-propagating action potentials.

Furthermore, the comparison of stochastic simulations with the experimental calcium transient constraints the number of ions released by the opening of RyRs; indeed, we find that releasing three to eight ions each time one of the 36 RyRs opens gives us a range of concentrations for experimental observations. Interestingly, we could recapitulate the decrease of released calcium by decreasing the released number from eight to six one-by-one, occurring once in a few milliseconds (Fig 5B). The distribution of RyRs has little influence on the calcium transient, as long as their interdistances are less than few tens of nanometers (see also S5 Fig). Note that we used here a minimum number of released calcium per RyR, but it is conceivable that more ions are indeed released. It would be quite interesting to obtain a direct measurement for the number of RyRs and the concentration of calcium release from RyRs located on the SA but also estimations of other parameters predicted by the present model such as the delay of calcium flux after activation and refractory periods.

### Role of extreme statistics in molecular transduction in nanodomains

As RyRs are activated by the two fastest ions that arrive to the binding sites, the physical separation of the initial calcium release in the spine head and the location of RyRs in the base is compensated by the redundancy based on the large initial number of released ions. The time scale induced by the fastest ions depends on the logarithm of initial ion number and the length of the direct ray, starting from the source and ending at the target [44].

The time scale generated by the fastest particle or ions is generic and can occur in many molecular transduction pathways in which there is a separation between the initial source and a second step that consists of amplifying the signal [45]. This is the case for second messengers such as IP3 [46], G-protein coupled receptors [47], and modulation of the inner hair cell voltage by CICR [48]. This time scale is very different from the Narrow Escape Time [39] phenomena, for which the time scale depends on the volume of the domain. We conclude that the statistics of the fastest particle compensates for long distances (limiting the importance of the neck length in calcium transmission, see S8 Fig), and the key modulating parameter is the number of initial ions.

### Can voltage-gated calcium channels contribute to fast calcium transient at the spine base?

Neuronal depolarization activates voltage-gated calcium channels (VGCCs) that result in calcium entry. In principle, calcium ions could enter in dendritic spines through VGCCs. However, little is known about their localization and whether or not they are present in the head of spines despite some studies reporting one to 20 channels [49]. No significant local effect of VGCCs was found to contribute to synaptic depolarization, and the amplitude of Ca^2+^ transients induced by excitatory postsynaptic potential seems to be regulated independent of VGCCs, as discussed in [50].

Under the hypothesis that VCGGs are located in the spine head, they would simply increase the number of initial calcium ions in the head but would not be implicated in accelerating a local transient at the base of the spine. If VGCCs are positioned on the membrane opposed to the RyRs located at the base, then a sufficient depolarization in the spine head would be needed, first to pass the high-neck voltage drop and second to activate VGCCs during synaptic activity. This scenario is unlikely, but it can exist independently of the extreme statistics diffusion that we have studied here. However, our glutamate- and calcium-uncaging experiments indicate that fast calcium increase at the base of a spine is unlikely to result from VGCC activation upon synaptic depolarization, as there would be no time delay compared to the calcium appearance in spine head, contrary to what we observe. To conclude, at this stage, we cannot rule out that in short spines, characterized by a low-neck resistance, RyRs open due to a sufficiently high membrane depolarization generated in the spine head. This scenario should be further investigated and may be relevant for short spines.

### Spontaneous calcium release from residual calcium

In dendrites, many buffers, ER–mitochondria interaction, calcium pumps, maintain the intracellular calcium at low concentration [4], but this concentration is subject to constant fluctuations. Small local fluctuations in the intracellular calcium concentration could result in spontaneous regenerative release events at the ER. In the model developed here, we wanted to estimate the rate of spontaneous regenerative release events. We assume that two calcium ions are necessary to trigger off a regenerative release from the surrounding cluster of RyRs, as long as the ER contains a sufficient amount of calcium ions. We recall that the distribution of residual calcium concentrations were found in neurons to be heterogenous, varying in the range 10–60 nM [51].

How does residual calcium activate CICR from RyRs? In a well-mixed dendritic subcompartment, the distribution of arrival time of an ion to a small channel site is Poissonian, described by *P*_1_(*t*) = λ*e*^−λ*t*^, in which the rate λ is the reciprocal of the mean first arrival time of a diffusing ion to a channel site [39]. The distribution of arrival times for two ions is computed as the convolution of the arrival time of the first with the second ion, leading to *P*_2_(*t*) = λ^2^*te*^−λ*t*^.

The mean time for the residual calcium to activate RyRs is equal to the mean time for two ions to find a target site, given by $\bar{\tau }=\int_{0}^{\infty }tP_{2}(t)dt=\frac{2}{\lambda }$. When there are *N* ions uniformly distributed in a subdendritic compartment, the mean duration between two calcium fluctuation events is thus the mean arrival time for two ions, chosen among *N*, that is *N*(*N* − 1)/2. We conclude that the mean time between two calcium release events when there are *N* ions is approximated by ${\bar{\tau }}_{N}=[2/N(N-1)]\bar{\tau }$, in which 1/*λ* = 100/(4*Da*) = 500 *s*, in which *V* = 100 *μm*^3^, *D* = = 20 *μm*^2^/*s* [52], and *a* = 10 *nm*. For a residual calcium concentration of 50 *nM*, the residual number of calcium is *N* ≈ 30 calcium and a mean duration between two consecutive calcium release events of the order of ${\underline{\tau }}_{N}\approx 1\;s$. This calcium increase is due to CICR from RyRs induced from the diffusion of residual calcium ions in the dendrite. When RyRs are hidden, this time between two spontaneous events could be exponentially longer [39], of the order of tens of seconds. To conclude, we found a time scale of calcium fluctuation of the order of few seconds when calcium ions are uniformly distributed at low concentration in dendrites < 50 *nM* [51]. If calcium ions are not uniformly distributed (out of equilibrium), a different approach should be considered. The residual calcium concentration is maintained low due to various types of calcium buffers, the presence of mitochondria below the spine neck, that could also regulate calcium concentration. How these processes maintain calcium locally low should certainly be further investigated.

### Consequences of amplifying calcium concentration at the base of a dendritic spine

What could be the role of calcium signal amplification induced by SA release at the base of a dendritic spine and not in the head? The asymmetry of RyR localization is a key feature in this difference, leaving the head compartment separated from the rest of the dendrite. Amplifying calcium at the base could favor receptor trafficking by influencing the delivery of AMPA receptors to dendritic spines. Successive calcium-accumulative events leading to SA refilling could trigger a massive release, while a depleted SA would only lead to a small release, suggesting an integrating role of the SA. Experimental evidences [53] further suggest that gating of the RyRs is also modulated by the luminal calcium concentration fluctuations, while the release can diminish when calcium is bound to buffers such as calsequestrin [54]. Another consequence of amplifying locally the calcium concentration at the ER is to trigger the production of ATP from nearby mitochondria [8]. Indeed, inducing ATP production requires that the calcium concentration reaches a threshold of 10 *μM*.

We studied here a time scale of a few to 20 ms. For longer durations, other mechanisms such as secondary messenger involving IP3 receptors [46] or the ORAI1 pathways [55] involved in SA replenishment can contribute to calcium concentration regulation. Future models should also incorporate the cycle of SA calcium, depletion using the ORAI1 pathways, and the calcium uptake at the base of the spine by mitochondria [56].

## Supporting information

S1 Data (XLSX)

S1 Fig Calibrating the model for the number of calcium pumps located in the spine head. (A) Simulation with calcium pumps uniformly distributed on the upper hemisphere of the spine surface. No SA is present (no contributions from RyRs and SERCA pumps). (B) Transient decay of the calcium number in the spine head (pumps are arranged as in [A]) when the number of pumps varies between *N* = 0 to *N* = 100 (the initial number of calcium ions is 1,000). We confirmed that *N* = 50 provides a matching approximation to the timescales that were obtained from calcium transient experiments. Therefore, we chose the value *N* = 50 for the remaining simulations. RyR, Ryanodyne receptor; SA, spine apparatus; SERCA, sarco/ER calcium-ATPase.
(PDF)

S2 Fig Calcium-dependent operation of RyRs. RyRs have been modeled in the past in the continuum limit description, as a boundary condition of the continuum reaction-diffusion equation [57]. However this condition cannot be used here in a stochastic approach. Instead, RyR opens in our stochastic simulations when 2 ions arrives at the catchment area of the receptor, which is a disk of radius *a* = 10 nm. This radius is comparable to the size revealed by crystallography studies [58, 59]. Note that fluctuations in the radius *a* = 10 nm are not expected to affect much the arrival time as shown by the formula for the first arrival, where the dependency in the radius *a* occurs through a log term, as shown analytically [44]. For simulating the RyR activity, we implemented the stochastic model [60]: when a first ion arrives from the cytoplasm, it is indefinitely bound to the RyR. When the second ion arrives at the same receptor, it opens the RyR, resulting an outflux of fixed number of calcium ions *n*_*Ca*_ (typically, *n*_*Ca*_ = 2 to 8, as mentioned in the figures of the main text) from the SA calcium stores to the cytosolic side of the spine. The number of released ions depends on the calcium concentration of ER and cytoplasm. Due to the unavailability of these values in literature, we used a total number of released ions from tens to few hundreds. These numbers are compatible with classical experiments where CICR leads to a fluctuation of calcium concentration with a magnitude of 100 nM [61]. A change of 100 nM is equivalent to 250 calcium ions in a volume of the size of the spine head. We assumed here that ER contains a sufficiently large amount of calcium ions and thus when around 300 ions are released though 36 receptors, we release around 8 ions per receptor. This number can decrease to zero when the ER does not contain calcium ions. Future research should investigate these predictions. RyR release is instantaneous, except in Fig 5B where we found upon testing several delays that a 0.25 ms delay is necessary for the simulations to agree with experimental calcium transient. The released calcium ions are placed at the center of the RyRs. This release is followed by a refractory period of a few milliseconds, as shown in each figure. If further ions arrive during this period, the receptor can bind maximum to one more (third) calcium ion. This situation corresponds to the third binding site proposed in [62]. For subsequent arrivals (*n* > 3), the receptor acts as a reflecting boundary. We arrange 36 RyRs at the base of the SA such that a third (12) of receptors is present on the top of the shaft, while the remaining two thirds (24) are distributed in four layers, each containing randomly-distributed six channels (Fig 4G). ER, endoplasmic reticulum; RyR, Ryanodyne receptor; SA, spine apparatus.
(PDF)

S3 Fig Calcium-dependent operation of SERCA pumps. We considered that calcium flow through SERCA pumps is unidirectional from the cytoplasmic side to the SA [63]. The pumps are opened by the arrival of two calcium ions to its binding sites from the cytoplasmic side [64]. Such opening event can translocate both calcium ions into the ER (luminal side). We modeled SERCA pumps as absorbing disks with a radius of 10 nm [65] with the precise operation as follows:
When the first calcium ion arrives at the circular disk of a pump from the cytoplasmic side, it is bound and retained for an indefinite time.When a second ion arrives at the SERCA pump from the cytoplasmic side, after binding of the first ion, the pump opens and both ions are moved into the SA.SERCA translocation time *τ*_*SER*,*TL*_ ≈ 100 *ms* is in the range of several hundred milliseconds [66], much longer than the total duration of our simulations which ran approximately 20 ms. Therefore, we consider the two ions to be indefinitely bound to the SERCA pump during the remaining duration of the simulation and no longer able to return to the spine.A SERCA pump is prevented from uptaking ions after the second ions is bound (step 2) and it is modeled in the simulation as a reflecting disk.
ER, endoplasmic retitculum; SA, spine apparatus; SERCA, sarco/ER calcium-ATPase.
(PDF)

S4 Fig Comparison of calcium transmission rates between SP+ve and SP−ve spines. The Calcium signal transmissions during uncaging experiments were compared as measurements from the focus of uncaging to the neighboring compartment, and the peak signal at uncaging is taken as 100%. (A) Transmission from the spine head to the dendrite and (B) from dendrite to the spine head. Left and right bars are the averaged transmission measures of SP+ and SP− spines. The focus of uncaging is marked with purple dot and the arrow indicates the possible signaling direction between the two compartments. Note much higher transmission rate from the head of SP+ spines to the dendrite, compared to dendrite to spine transmission (A: *t* probability < 0.0001). The rate of transmission in both directions for the SP− spine is the same (B: *t* probability 0.6). Here, the number of spines is ***N*** = **3** for SP+ and **7** for SP−. This result confirms that the presence of a spine apparatus is critical for an effective uni-directional calcium flow in the spine. (C) In a set of control experiments, NP-EGTA was replaced with caged fluorescein, a biologically neutral molecule that becomes fluorescent only after its flash photolysis. The rate of transmission from spine head of SP+ and SP− spines to their parent dendrites in this case had no significant statistical difference, while the lengths of the spines were approximately the same length as in A and B. The slightly smaller percentages found for fluorescein here (compared to calcium in A and B) can probably be attributed to the larger mass of fluorescein molecules. SP, synaptopodin.
(PDF)

S5 Fig Impact of inter-RyR distance on calcium induced calcium release. (A) Twenty RyRs are positioned at the base of the ER neck surface and arranged in a grid with an inter-RyR distance (*d*_RyR_), between 26 to 156 nm. (B) One thousand calcium ions are released from the center of the spine head, and we estimated from numerical simulations the cumulative sum of calcium ions arriving in the base of the dendrite (similar to Fig 4A and 4B) compared to the control (orange), in which no calcium ions are released from the RyRs but can only arrive from the head. There is a slight reduction in the number of calcium arriving at the base only when the inter-RyR distance *d*_*RyR*_ was increased to 156 nm. Therefore, we confirm that in the range of the present simulations, the reported amplifications of calcium signal do not depend on the distance among neighboring RyRs. ER, endoplasmic reticulum; RyR, Ryanodyne receptor.
(PDF)

S6 Fig Simulating the dependency of CICR on ER calcium concentration. Calcium concentration in the spine apparatus can decay each time a RyR opens. We tested four different configurations of RyR positioning ([A–D] with three different initial numbers of calcium in the SA *N* = 50, 150, and 500 [E–G]). (Initial calcium release occurs at the center of the spine head, similar to Fig 3D and each curve was obtained using 20 runs.) When there is a smaller number of calcium ions (50 or 150), calcium increase at the base is limited to the case when RyRs are placed only at the base of the SA (red curves). Calcium decay in the SA (left plots) shows that this is due to the RyRs located in the SA head being triggered too quickly by the calcium ions arriving from the nearby uncaging spot, leading to a fast drop in calcium concentration in the SA. This release leaves SA with insufficient calcium ions to generate a large CICR response when calcium ions arrive at the base; therefore, calcium at the base is limited to the few ions arriving from the head. In that case, the mechanism of amplification is almost abolished (flat curves). The present results confirm our experimental and simulation results (Fig 5), showing that the calcium response was amplified at the base only when RyRs were located at this same place. When there are enough calcium ions (*N* = 500 ions in G), having RyRs lead to calcium depletion in the ER, but there is still enough calcium in the SA to elicit a response at the base (blue and green). However, the optimal positioning of RyRs to trigger the strongest response remains at the base (red curve). We conclude that for the compatibility between glutamate- and calcium-uncaging experiments and stochastic simulations with limited and unlimited calcium, it require a RyR distribution enriched at the base of dendritic spines. CICR, calcium-induced calcium release; ER, endoplasmic reticulum; RyR, Ryanodyne receptor; SA, spine apparatus.
(PDF)

S7 FigRelease dynamics from RyRs induced by cytoplasmic calcium ions.Histograms of the release times of RyRs in the three consecutive release events shown in Fig 5B. The number of release ions decrease starting from *N* = 8, then to 7 and finally to 6. (36 RyR receptors are positioned on the SA and 25 trials were run). The interval between each release is 3 ms, and after two calcium ions have arrived, it takes 0.25 ms for a RyR to open and release RyR. We conclude that calcium release occurs in wave packets, with each release leading to the release of new ions that can open the neighbouring RyRs and thus leading to CICR. Moreover, this simulation confirmed that all RyRs do participate to this process, regardless of the distances among them. The mean release times $\bar{\tau }$ shown in Fig 5B (green arrows) were evaluated using these three histograms. CICR, calcium-induced calcium release; RyR, Ryanodyne receptor; SA, spine apparatus.(PDF)

S8 Fig Dependence of extreme statistics mechanism on the length of spine necks. Arrival time distributions for three different spine neck lengths (L = 1 **μ**m, 1.5 **μ**m, and 2 **μ**m), modeled as a one dimensional segment with a reflecting boundary at the origin x = 0 and absorption at x = L. Here, *N* = 5 ions were used to match the very small number of ions that escapes the spine head and remains in the neck during the first few milliseconds. The mean arrival times for the first two ions in the three neck lengths are 0.49 ms, 1.06 ms, and 1.85 ms, confirming that uncaging location and the spine neck length have little impact on the time course of calcium release from the ER. These simulations confirm the associated experimental results in dendritic spine, based on glutamate uncaging [67]. In general three dimensional geometry, trajectories associated to the fastest particles are concentrated near the shortest path, therefore changing the initial calcium injection location within the spine head does not change much the delay of calcium induced calcium release [68]. ER, endoplasmic reticulum.
(PDF)

S1 Table Parameters for calcium transients. (PDF)

S2 Table Number of trials for numerical simulation results. (PDF)

S1 Text Mathematical derivations of arrival times of the first and second ions. (PDF)

## Abbreviations

AMPA: α-amino-3-hydroxy-5-methyl-4- isoxazolepropionic acid
CICR: calcium-induced calcium release
ER: endoplasmic reticulum
GluR-1: glutamate receptor 1
RyR: Ryanodyne receptor
SA: spine apparatus
SERCA: sarco/ER calcium-ATPase
SP: synaptopodin
STED: stimulated emission depletion
VGCC: voltage-gated calcium channel
