# Correction: Fast calcium transients in dendritic spines driven by extreme statistics

**DOI:** 10.1371/journal.pbio.3001663

**Published:** 2022-05-27

**Authors:** Kanishka Basnayake, David Mazaud, Alexis Bemelmans, Nathalie Rouach, Eduard Korkotian, David Holcman

After this article [1] was published, concerns were raised about some of the reported results and about the article’s adherence to the PLOS Data Availability policy. *PLOS Biology* investigated the issues raised and concluded that the article as published is scientifically valid but data and code availability updates are needed to bring the article into compliance with journal policies.

As written in the original article, the codes and underlying data for this study [1], including data supporting simulation results, are available at http://bionewmetrics.org/simulations-of-spine-calcium-transients-with-er/.

In S1 File, the data for panels 1B and 1C are reported with a time resolution of 0.1 ms, when shifted by the uncaging flash arrival time (*t* = 0) between 3.6 to 3.8 ms (depending on the scanner position). The number of rows for each dataset corresponds to recording duration, which was 26 and 27 milliseconds for SP- and SP+ spines, respectively.

The figure legend for S4 Fig in [1] provides incorrect *n* values for the experiments shown in panels A and B. The correct numbers of spines represented in this figure are as follows: (A, B) *n* = 19 for SP+ and *n* = 27 for SP-; (C) *n* = 3 for SP+ and *n* = 7 for SP-. (See S1 Data [1] for the full dataset.)

There is a labelling error in the published S1 Data file [1]: the data reported in the last tab is for S4 Fig (not S1 Fig).

## Supporting information

S1 File Data for panels Fig 1A-C. (XLSX)
