# Supplementary figures and images for: Fast calcium transients in dendritic spines driven by extreme statistics
Source: PLoS Biol. 2019 Jun 4;17(6):e2006202. doi: 10.1371/journal.pbio.2006202 (PMC6548358; doi:10.1371/journal.pbio.2006202)

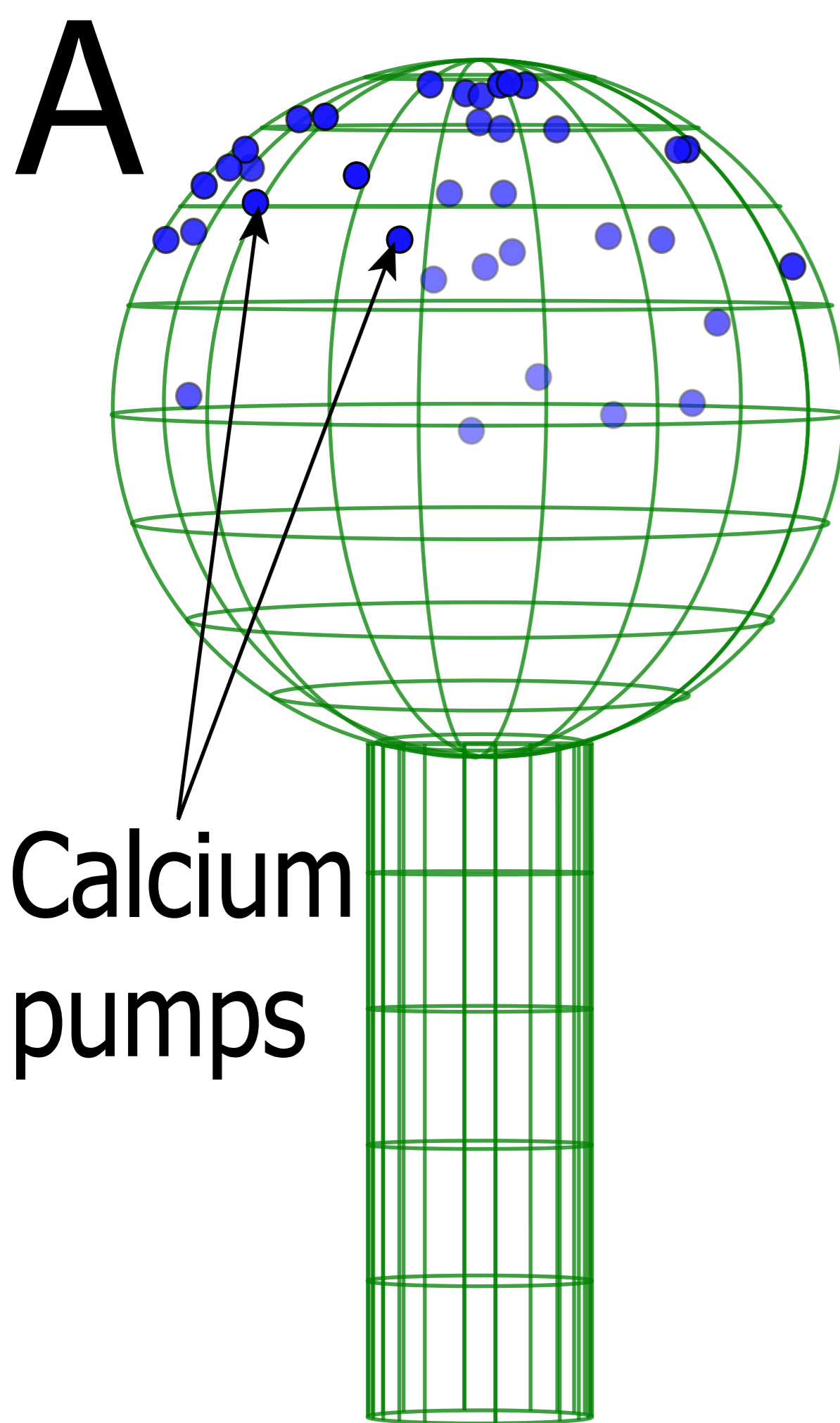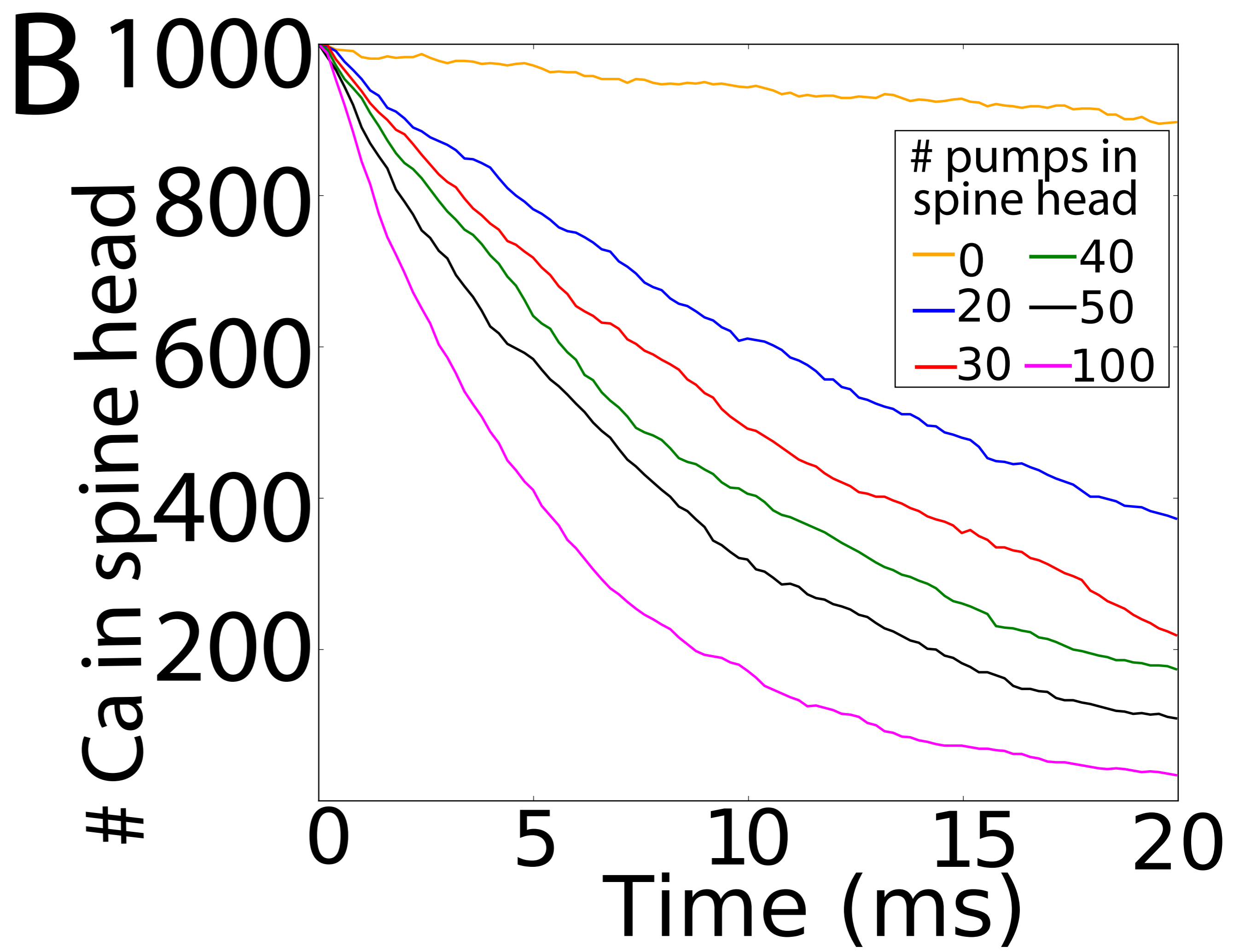

Supplement: S1 Fig — (A) Simulation with calcium pumps uniformly distributed on the upper hemisphere of the spine surface. No SA is present (no contributions from RyRs and SERCA pumps). (B) Transient decay of the calcium number in the spine head (pumps are arranged as in [A]) when the number of pumps varies between N = 0 to N = 100 (the initial number of calcium ions is 1,000). We confirmed that N = 50 provides a matching approximation to the timescales that were obtained from calcium transient experiments. Therefore, we chose the value N = 50 for the remaining simulations. RyR, Ryanodyne receptor; SA, spine apparatus; SERCA, sarco/ER calcium-ATPase. (PDF) [file pbio.2006202.s002.pdf]

Closed

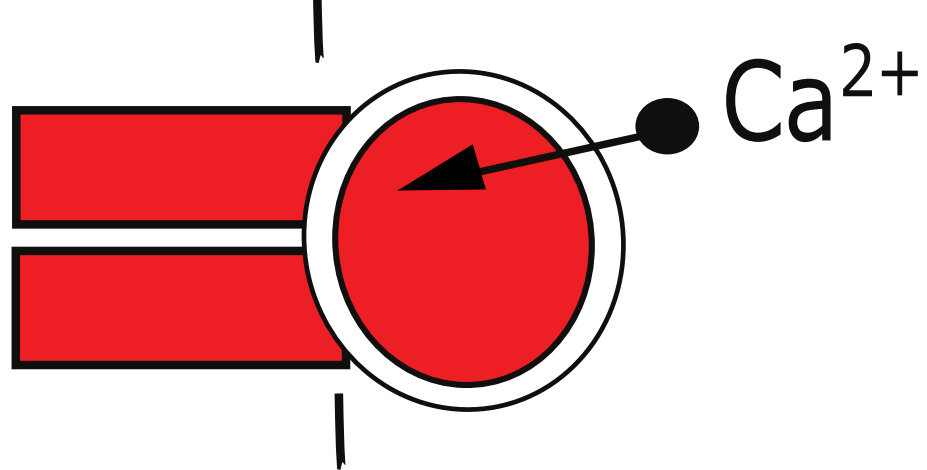

Bound

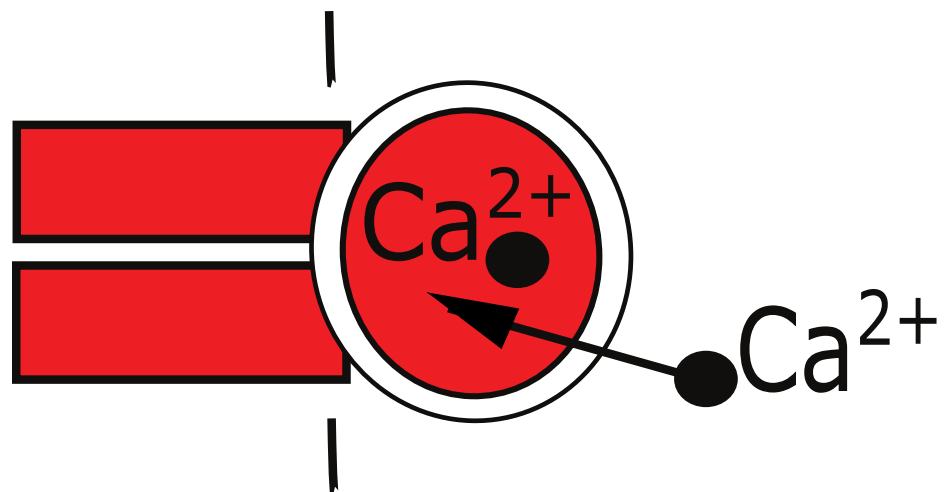

Open

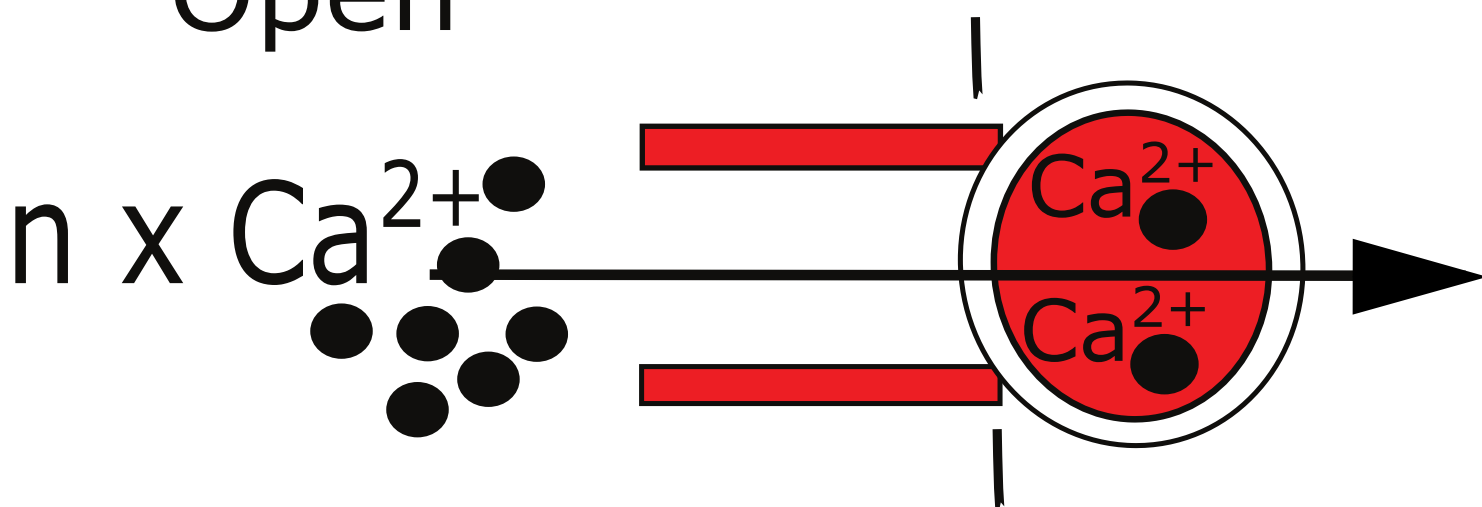

Release

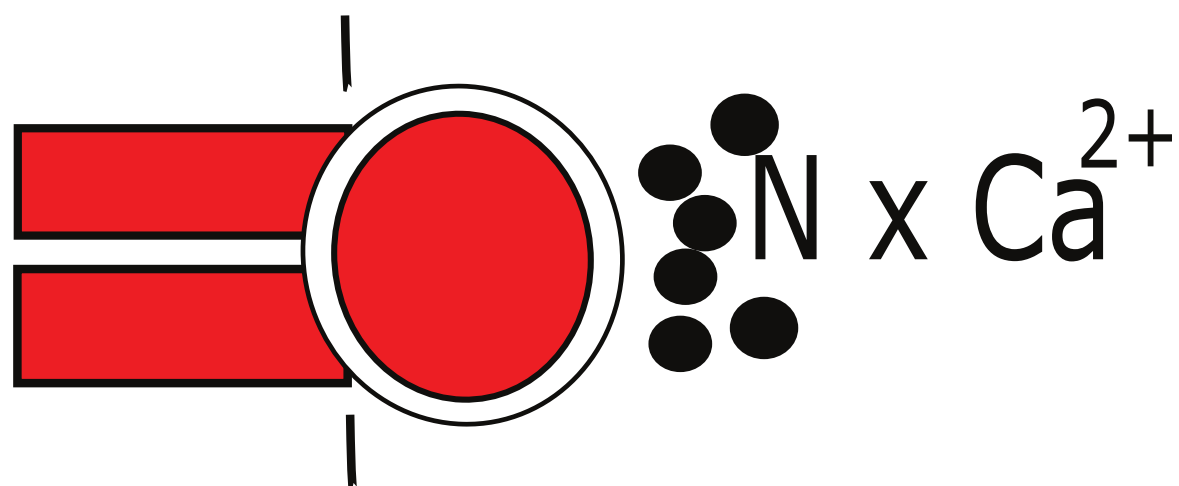

Supplement: S2 Fig — RyRs have been modeled in the past in the continuum limit description, as a boundary condition of the continuum reaction-diffusion equation [57]. However this condition cannot be used here in a stochastic approach. Instead, RyR opens in our stochastic simulations when 2 ions arrives at the catchment area of the receptor, which is a disk of radius a = 10 nm. This radius is comparable to the size revealed by crystallography studies [58, 59]. Note that fluctuations in the radius a = 10 nm are not expected to affect much the arrival time as shown by the formula for the first arrival, where the dependency in the radius a occurs through a log term, as shown analytically [44]. For simulating the RyR activity, we implemented the stochastic model [60]: when a first ion arrives from the cytoplasm, it is indefinitely bound to the RyR. When the second ion arrives at the same receptor, it opens the RyR, resulting an outflux of fixed number of calcium ions nCa (typically, nCa = 2 to 8, as mentioned in the figures of the main text) from the SA calcium stores to the cytosolic side of the spine. The number of released ions depends on the calcium concentration of ER and cytoplasm. Due to the unavailability of these values in literature, we used a total number of released ions from tens to few hundreds. These numbers are compatible with classical experiments where CICR leads to a fluctuation of calcium concentration with a magnitude of 100 nM [61]. A change of 100 nM is equivalent to 250 calcium ions in a volume of the size of the spine head. We assumed here that ER contains a sufficiently large amount of calcium ions and thus when around 300 ions are released though 36 receptors, we release around 8 ions per receptor. This number can decrease to zero when the ER does not contain calcium ions. Future research should investigate these predictions. RyR release is instantaneous, except in Fig 5B where we found upon testing several delays that a 0.25 ms delay is necessary for the simulati [file pbio.2006202.s003.pdf]

Closed

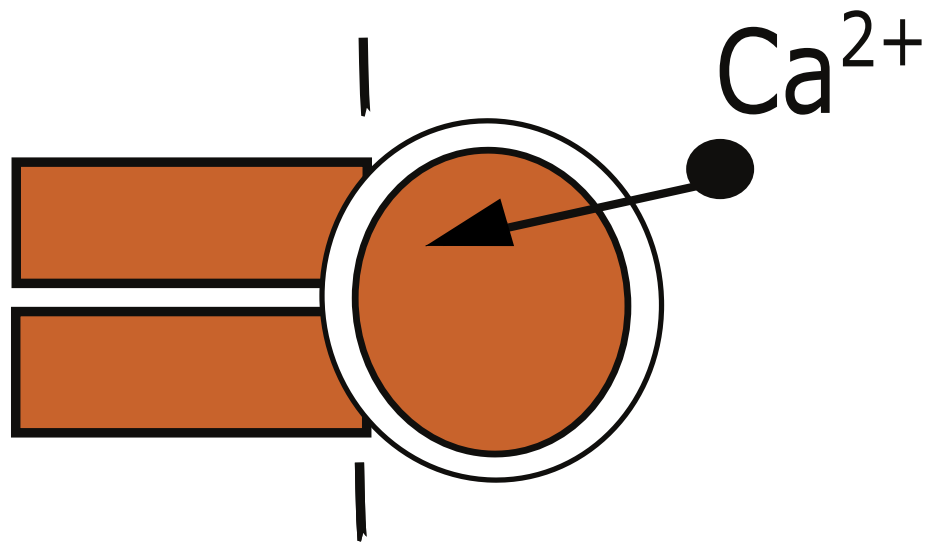

Bound

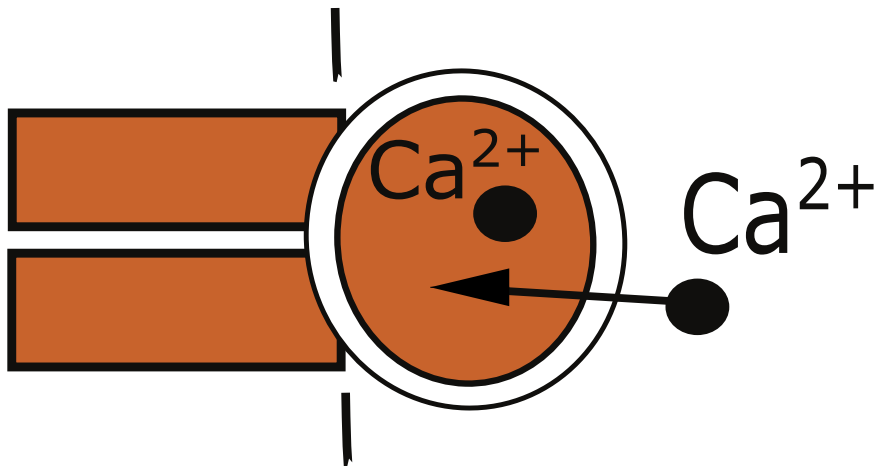

Open

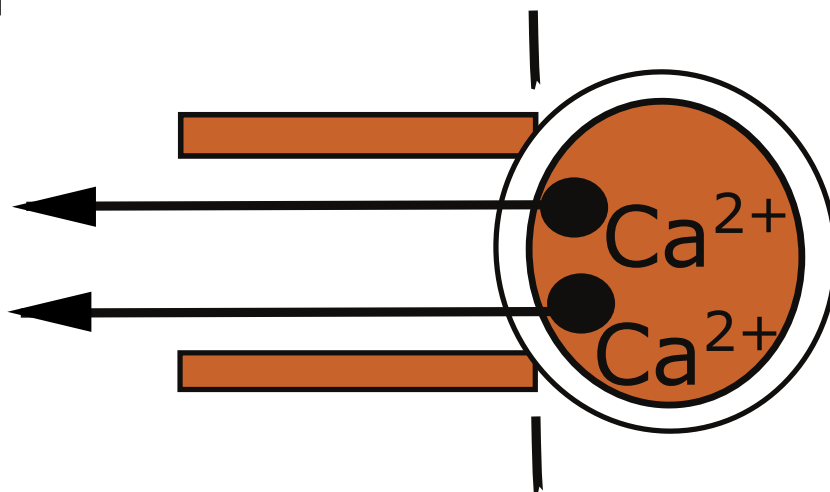

Translocation

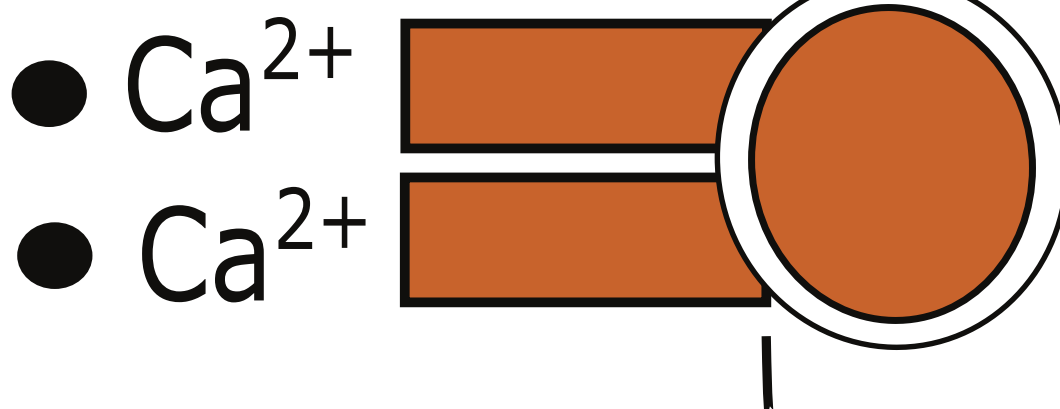

Supplement: S3 Fig — We considered that calcium flow through SERCA pumps is unidirectional from the cytoplasmic side to the SA [63]. The pumps are opened by the arrival of two calcium ions to its binding sites from the cytoplasmic side [64]. Such opening event can translocate both calcium ions into the ER (luminal side). We modeled SERCA pumps as absorbing disks with a radius of 10 nm [65] with the precise operation as follows: When the first calcium ion arrives at the circular disk of a pump from the cytoplasmic side, it is bound and retained for an indefinite time.When a second ion arrives at the SERCA pump from the cytoplasmic side, after binding of the first ion, the pump opens and both ions are moved into the SA.SERCA translocation time τSER,TL ≈ 100 ms is in the range of several hundred milliseconds [66], much longer than the total duration of our simulations which ran approximately 20 ms. Therefore, we consider the two ions to be indefinitely bound to the SERCA pump during the remaining duration of the simulation and no longer able to return to the spine.A SERCA pump is prevented from uptaking ions after the second ions is bound (step 2) and it is modeled in the simulation as a reflecting disk. ER, endoplasmic retitculum; SA, spine apparatus; SERCA, sarco/ER calcium-ATPase. (PDF) [file pbio.2006202.s004.pdf]

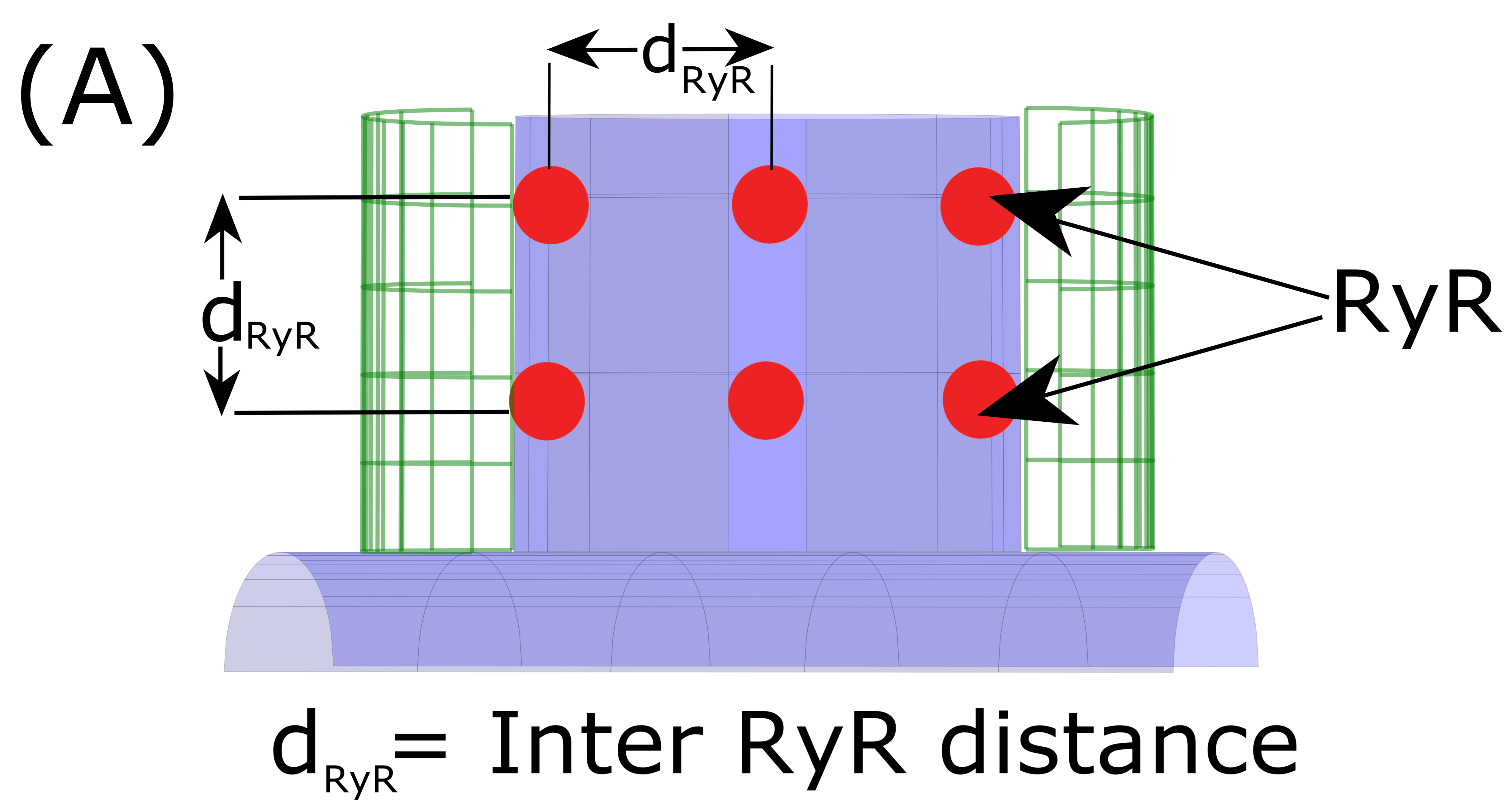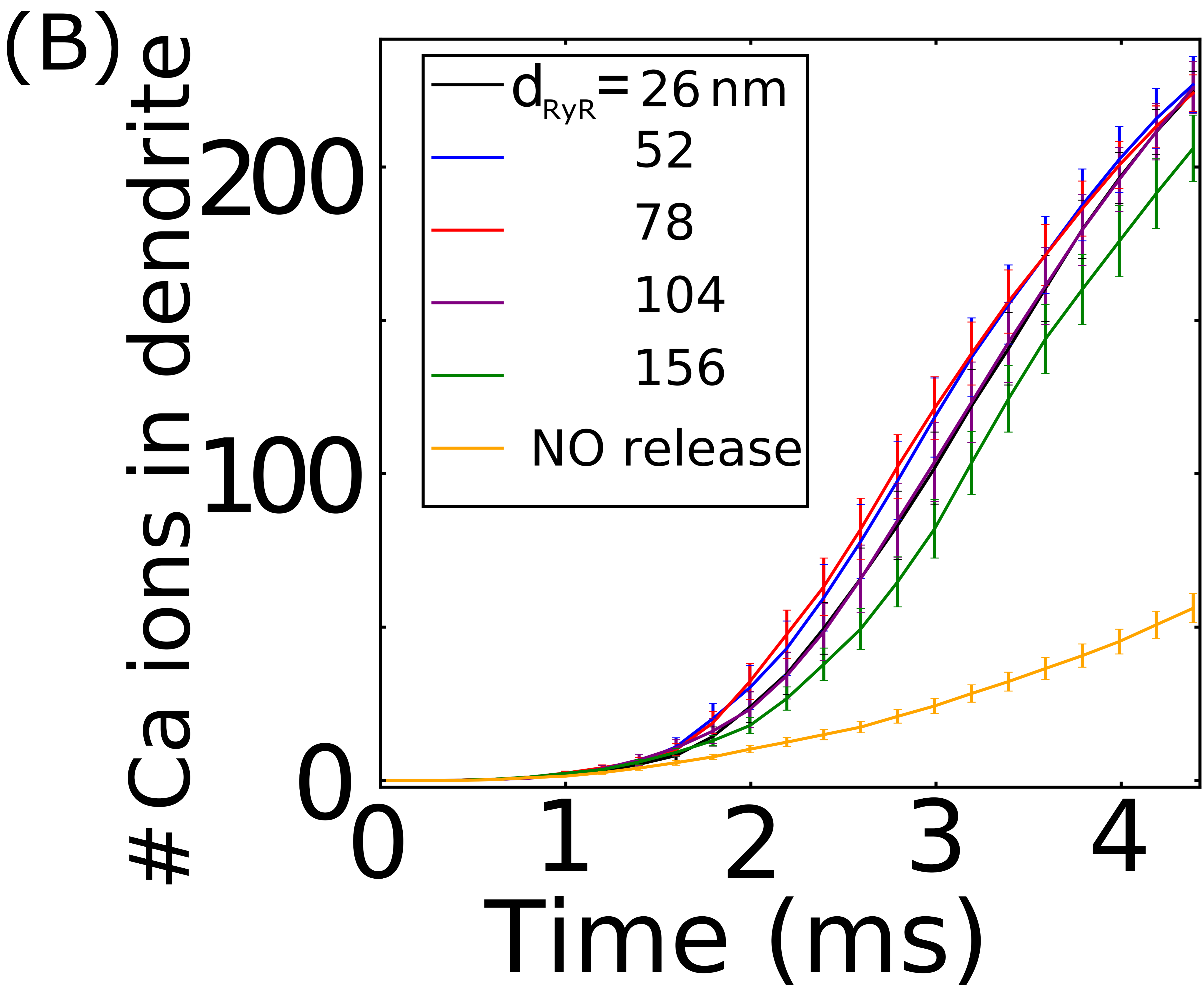

Supplement: S5 Fig — (A) Twenty RyRs are positioned at the base of the ER neck surface and arranged in a grid with an inter-RyR distance (dRyR), between 26 to 156 nm. (B) One thousand calcium ions are released from the center of the spine head, and we estimated from numerical simulations the cumulative sum of calcium ions arriving in the base of the dendrite (similar to Fig 4A and 4B) compared to the control (orange), in which no calcium ions are released from the RyRs but can only arrive from the head. There is a slight reduction in the number of calcium arriving at the base only when the inter-RyR distance dRyR was increased to 156 nm. Therefore, we confirm that in the range of the present simulations, the reported amplifications of calcium signal do not depend on the distance among neighboring RyRs. ER, endoplasmic reticulum; RyR, Ryanodyne receptor. (PDF) [file pbio.2006202.s006.pdf]

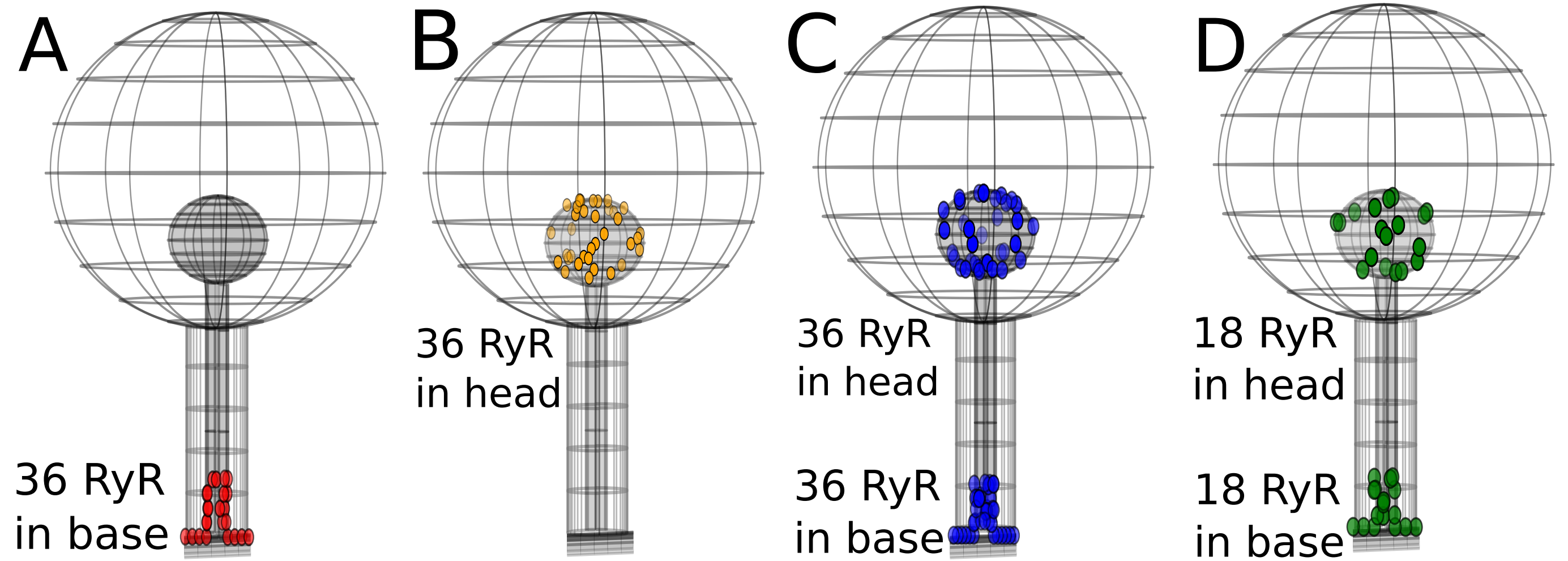

# initial Calcium = 50

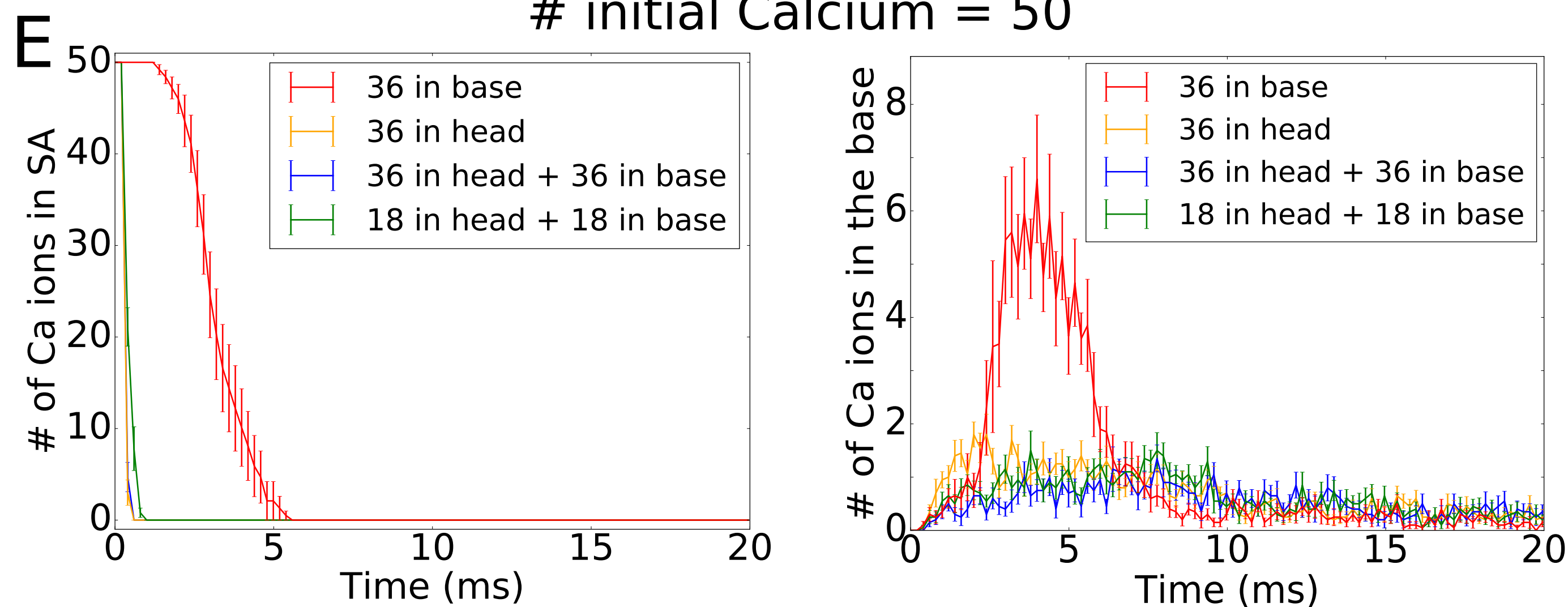

# initial Calcium = 150

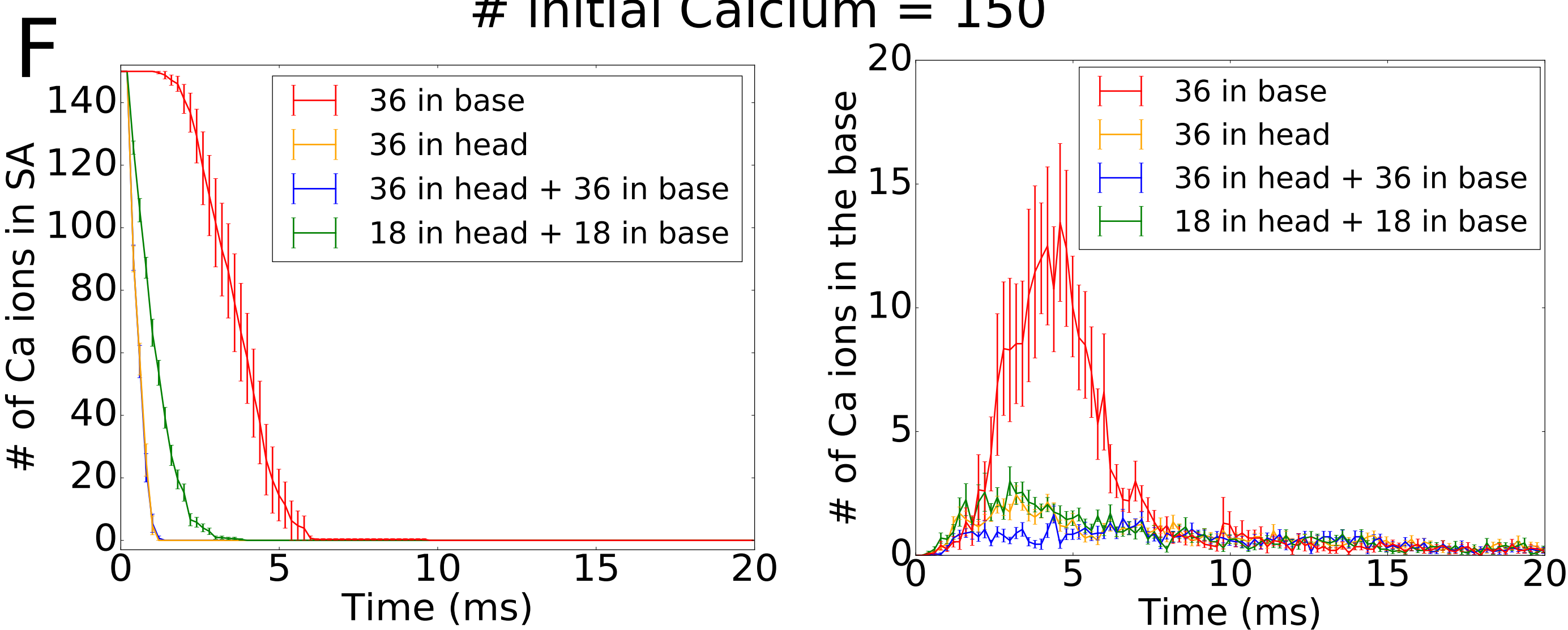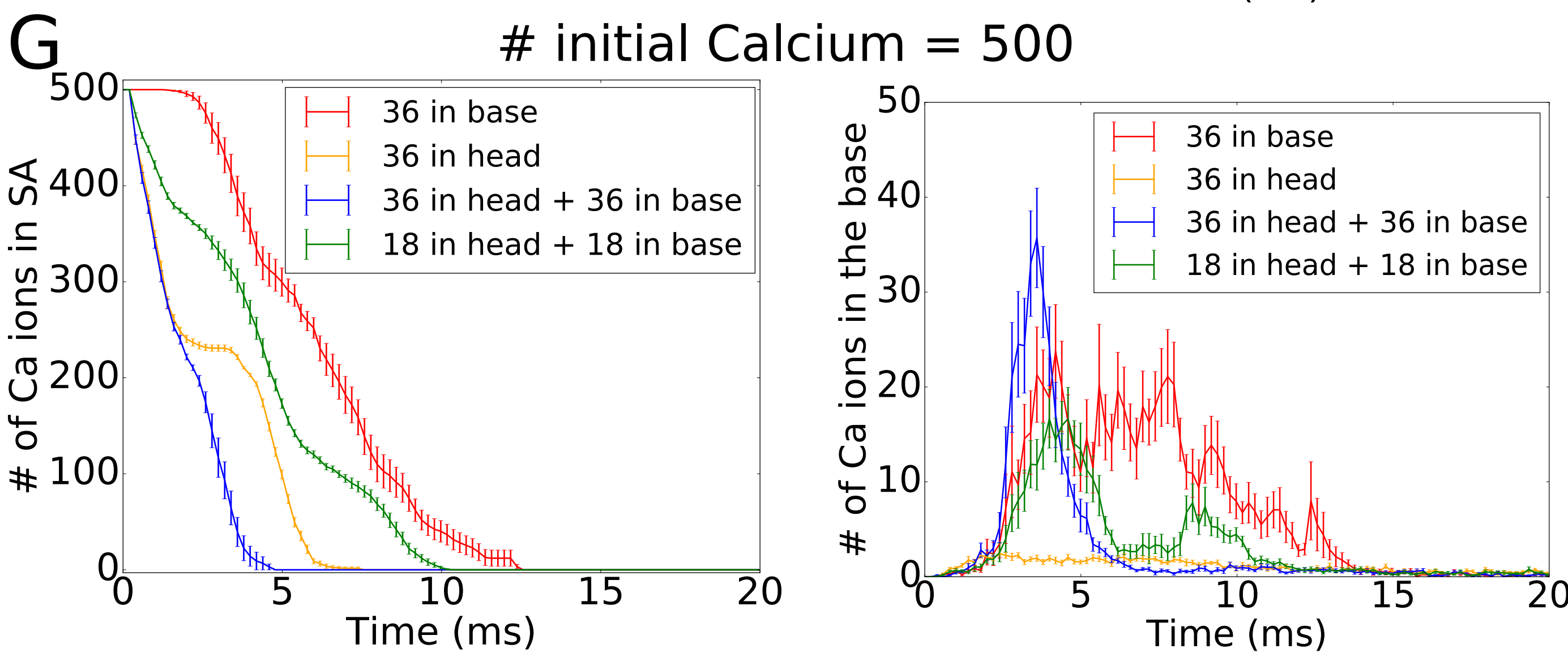

Supplement: S6 Fig — Calcium concentration in the spine apparatus can decay each time a RyR opens. We tested four different configurations of RyR positioning ([A–D] with three different initial numbers of calcium in the SA N = 50, 150, and 500 [E–G]). (Initial calcium release occurs at the center of the spine head, similar to Fig 3D and each curve was obtained using 20 runs.) When there is a smaller number of calcium ions (50 or 150), calcium increase at the base is limited to the case when RyRs are placed only at the base of the SA (red curves). Calcium decay in the SA (left plots) shows that this is due to the RyRs located in the SA head being triggered too quickly by the calcium ions arriving from the nearby uncaging spot, leading to a fast drop in calcium concentration in the SA. This release leaves SA with insufficient calcium ions to generate a large CICR response when calcium ions arrive at the base; therefore, calcium at the base is limited to the few ions arriving from the head. In that case, the mechanism of amplification is almost abolished (flat curves). The present results confirm our experimental and simulation results (Fig 5), showing that the calcium response was amplified at the base only when RyRs were located at this same place. When there are enough calcium ions (N = 500 ions in G), having RyRs lead to calcium depletion in the ER, but there is still enough calcium in the SA to elicit a response at the base (blue and green). However, the optimal positioning of RyRs to trigger the strongest response remains at the base (red curve). We conclude that for the compatibility between glutamate- and calcium-uncaging experiments and stochastic simulations with limited and unlimited calcium, it require a RyR distribution enriched at the base of dendritic spines. CICR, calcium-induced calcium release; ER, endoplasmic reticulum; RyR, Ryanodyne receptor; SA, spine apparatus. (PDF) [file pbio.2006202.s007.pdf]

# release:  $n_{Ca}=8$

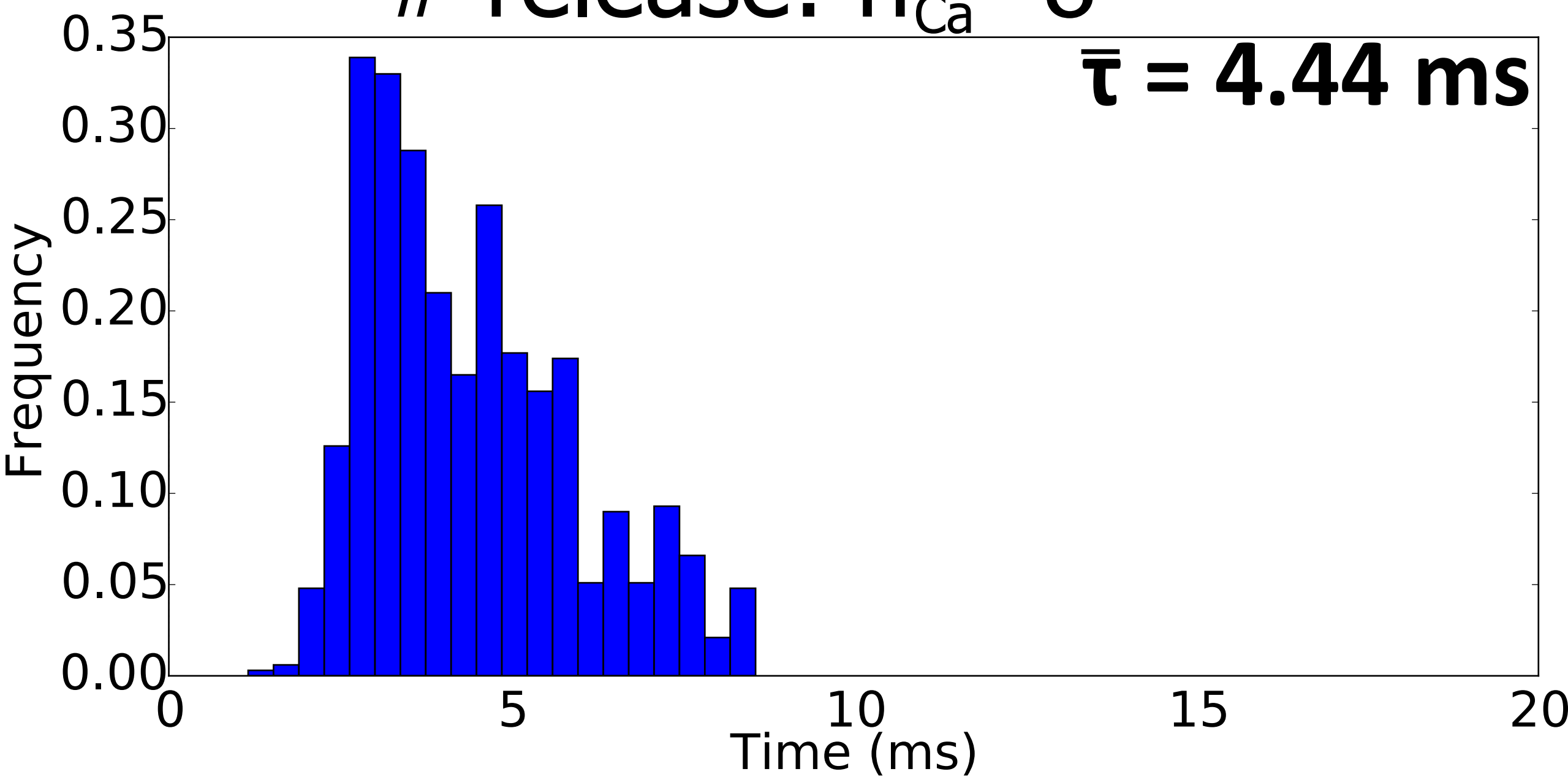

$n_{Ca} = 7$

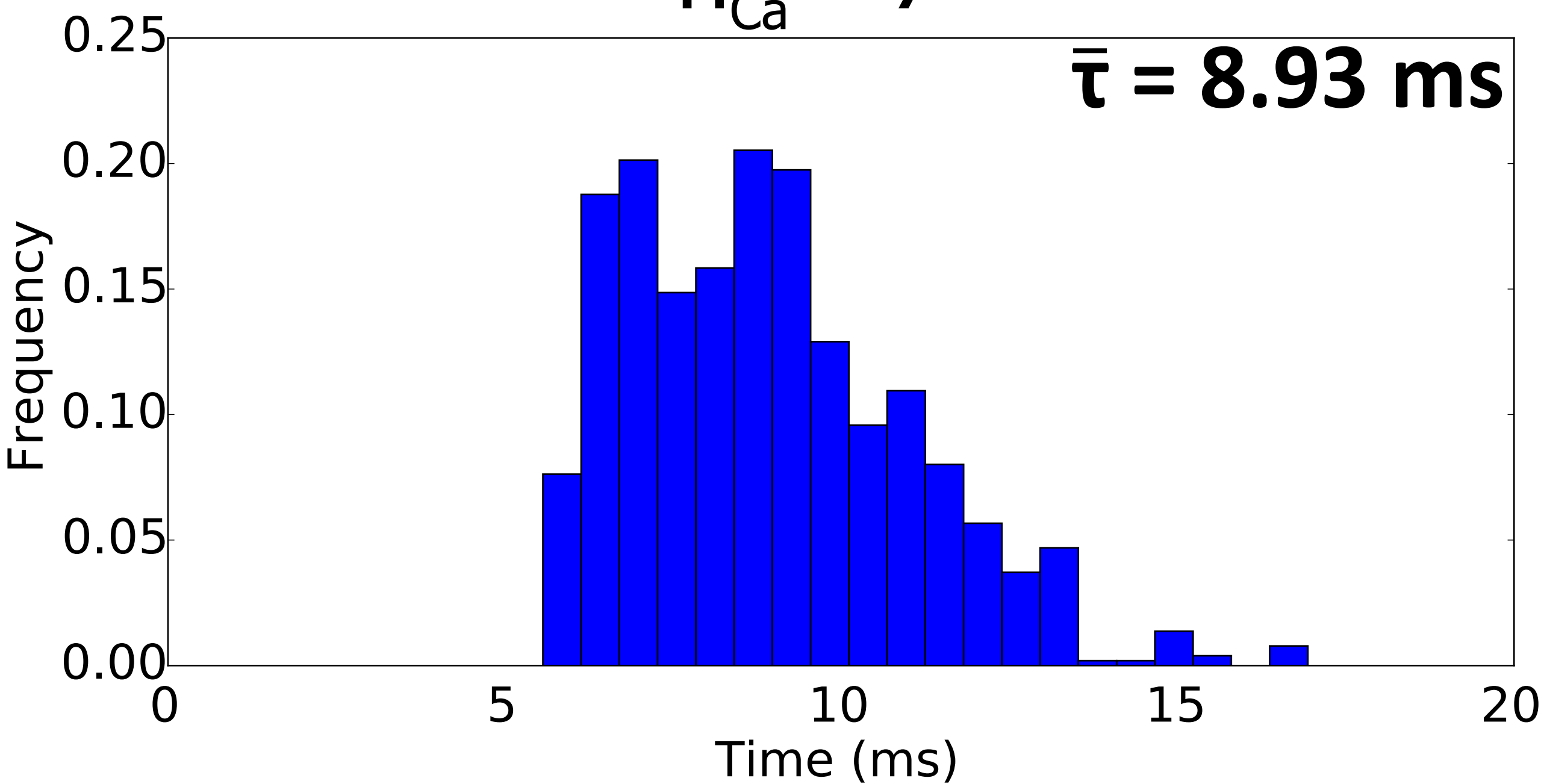

$n_{Ca} = 6$

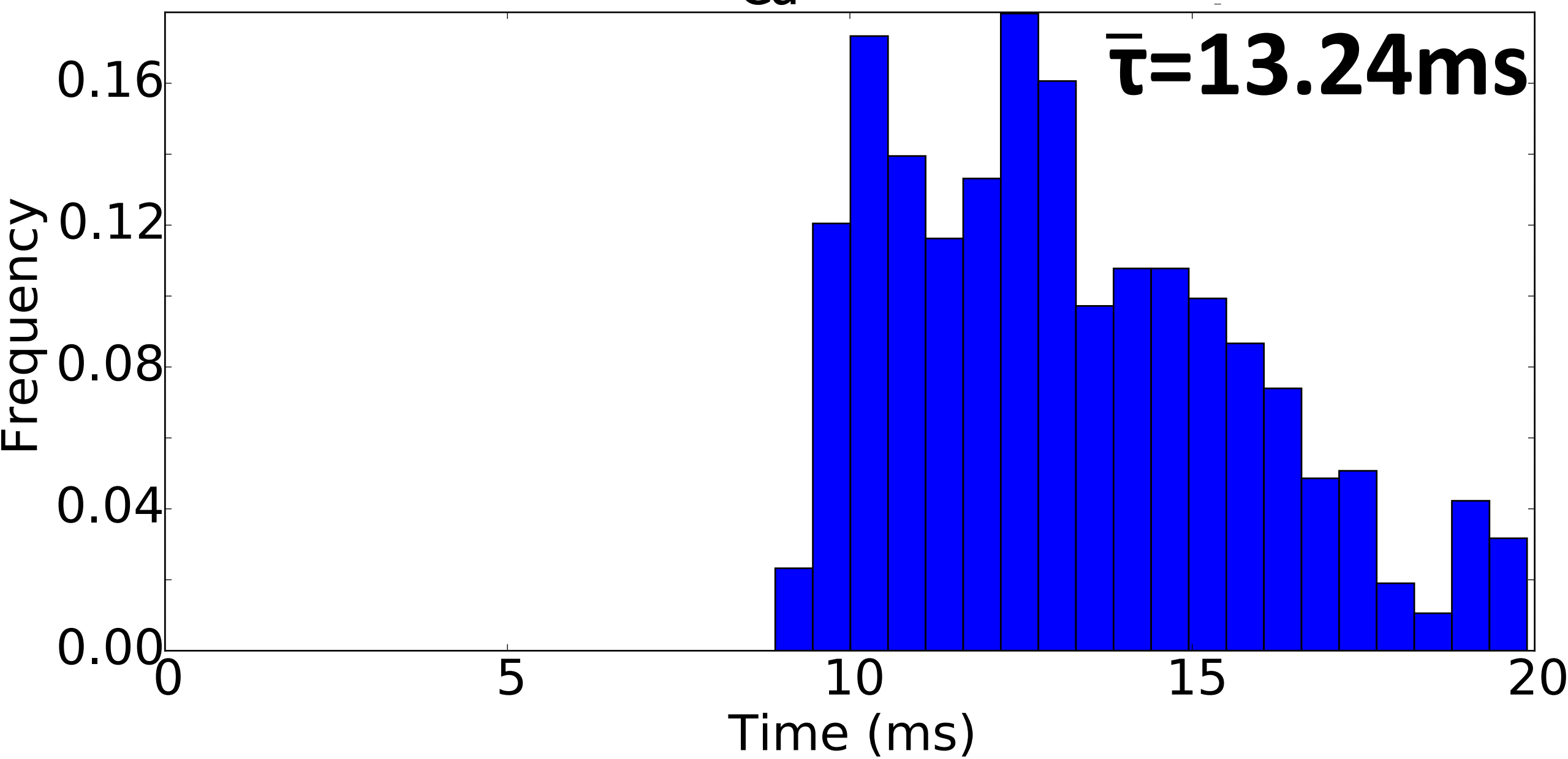

Supplement: S7 Fig — Histograms of the release times of RyRs in the three consecutive release events shown in Fig 5B. The number of release ions decrease starting from N = 8, then to 7 and finally to 6. (36 RyR receptors are positioned on the SA and 25 trials were run). The interval between each release is 3 ms, and after two calcium ions have arrived, it takes 0.25 ms for a RyR to open and release RyR. We conclude that calcium release occurs in wave packets, with each release leading to the release of new ions that can open the neighbouring RyRs and thus leading to CICR. Moreover, this simulation confirmed that all RyRs do participate to this process, regardless of the distances among them. The mean release times τ¯ shown in Fig 5B (green arrows) were evaluated using these three histograms. CICR, calcium-induced calcium release; RyR, Ryanodyne receptor; SA, spine apparatus. (PDF) [file pbio.2006202.s008.pdf]

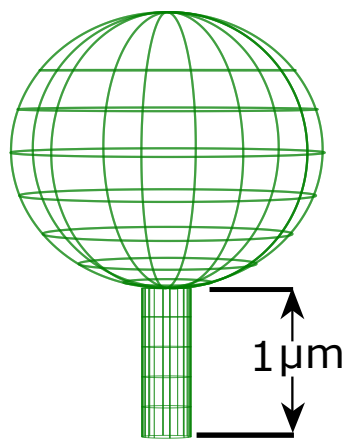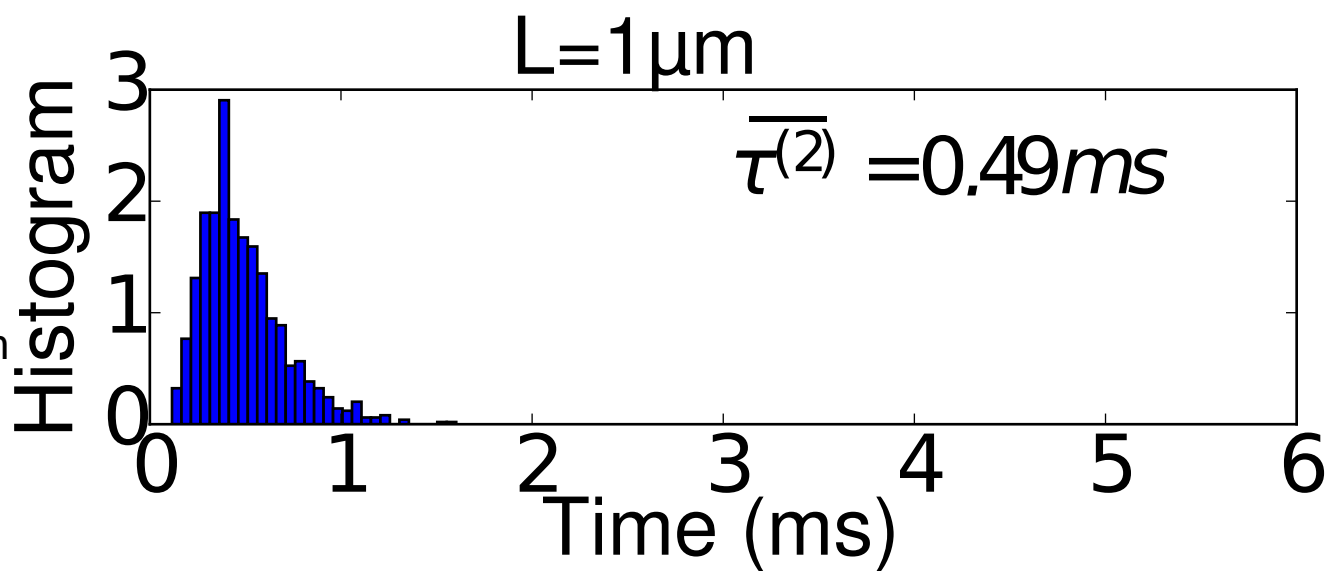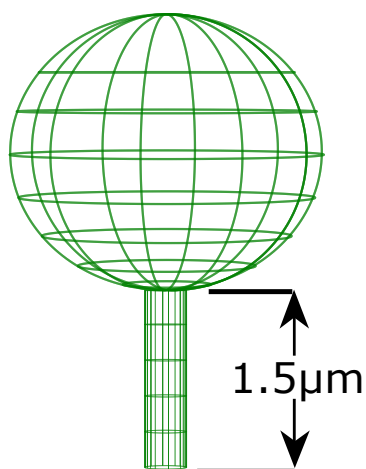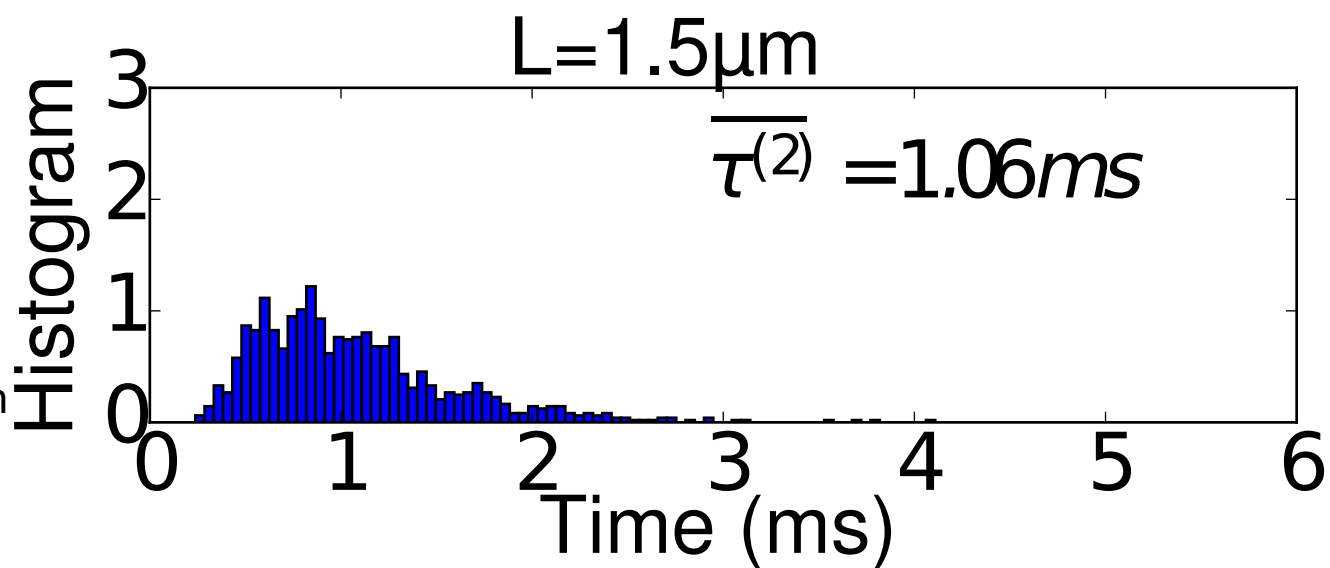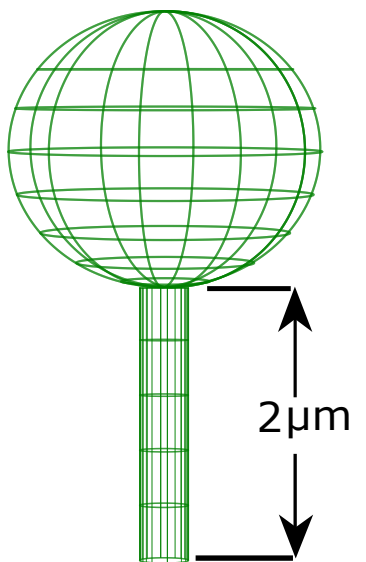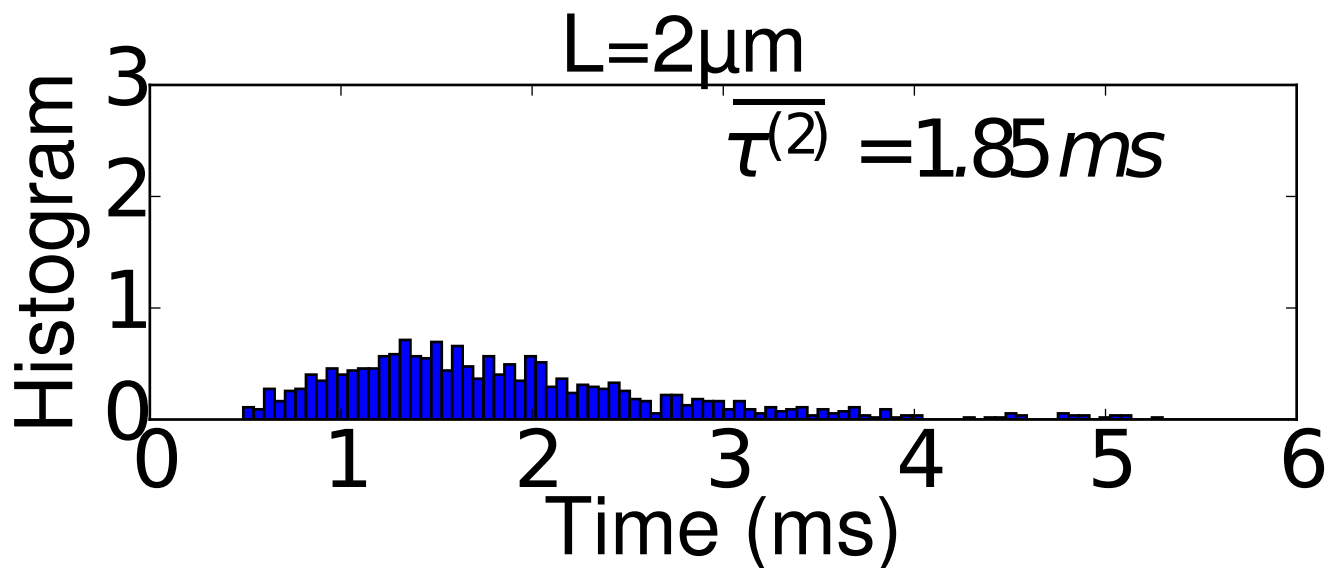

Supplement: S8 Fig — Arrival time distributions for three different spine neck lengths (L = 1 μm, 1.5 μm, and 2 μm), modeled as a one dimensional segment with a reflecting boundary at the origin x = 0 and absorption at x = L. Here, N = 5 ions were used to match the very small number of ions that escapes the spine head and remains in the neck during the first few milliseconds. The mean arrival times for the first two ions in the three neck lengths are 0.49 ms, 1.06 ms, and 1.85 ms, confirming that uncaging location and the spine neck length have little impact on the time course of calcium release from the ER. These simulations confirm the associated experimental results in dendritic spine, based on glutamate uncaging [67]. In general three dimensional geometry, trajectories associated to the fastest particles are concentrated near the shortest path, therefore changing the initial calcium injection location within the spine head does not change much the delay of calcium induced calcium release [68]. ER, endoplasmic reticulum. (PDF) [file pbio.2006202.s009.pdf]
