# Supplementary material for: Fast calcium transients in dendritic spines driven by extreme statistics
Source: PLoS Biol. 2019 Jun 4;17(6):e2006202. doi: 10.1371/journal.pbio.2006202 (PMC6548358; doi:10.1371/journal.pbio.2006202)
Supplement: S4 Fig — The Calcium signal transmissions during uncaging experiments were compared as measurements from the focus of uncaging to the neighboring compartment, and the peak signal at uncaging is taken as 100%. (A) Transmission from the spine head to the dendrite and (B) from dendrite to the spine head. Left and right bars are the averaged transmission measures of SP+ and SP− spines. The focus of uncaging is marked with purple dot and the arrow indicates the possible signaling direction between the two compartments. Note much higher transmission rate from the head of SP+ spines to the dendrite, compared to dendrite to spine transmission (A: t probability < 0.0001). The rate of transmission in both directions for the SP− spine is the same (B: t probability 0.6). Here, the number of spines is N = 3 for SP+ and 7 for SP−. This result confirms that the presence of a spine apparatus is critical for an effective uni-directional calcium flow in the spine. (C) In a set of control experiments, NP-EGTA was replaced with caged fluorescein, a biologically neutral molecule that becomes fluorescent only after its flash photolysis. The rate of transmission from spine head of SP+ and SP− spines to their parent dendrites in this case had no significant statistical difference, while the lengths of the spines were approximately the same length as in A and B. The slightly smaller percentages found for fluorescein here (compared to calcium in A and B) can probably be attributed to the larger mass of fluorescein molecules. SP, synaptopodin. (PDF) [file pbio.2006202.s005.pdf]

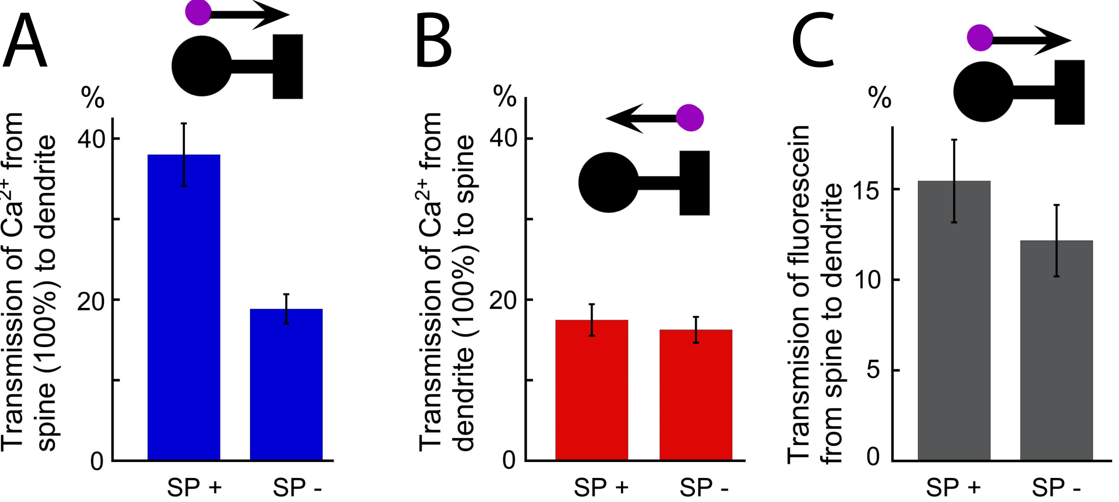

A
%
Transmission of Ca^{2+} from spine (100%) to dendrite
40
20
0
SP +
SP -
B
%
Transmission of Ca^{2+} from dendrite (100%) to spine
40
20
0
SP +
SP -
C
%
Transmision of fluorescein from spine to dendrite
15
10
5
0
SP +
SP -
