# Supplementary material for: Fast calcium transients in dendritic spines driven by extreme statistics
Source: PLoS Biol. 2019 Jun 4;17(6):e2006202. doi: 10.1371/journal.pbio.2006202 (PMC6548358; doi:10.1371/journal.pbio.2006202)
Supplement: S1 Table — (PDF) [file pbio.2006202.s010.pdf]

Table S1: Parameters for calcium transient

Parameters	Symbols	Values
Time step	Δt	10^{-7} s
Diffusion	D	600 $\mu m^2 s^{-1}$ [37]
Initial number of calcium ions	N	1000
Spine head radius	R	1 μm
Spine neck radius	a	0.15 μm
Spine neck length	L	1.5 μm
SA head radius	R_{SA}	0.25 μm
SA neck length	L_{SA}	1.5 μm
SA neck radius	a_{SA}	0.05 μm
Spine head-SA head center distance	l	0.5 μm
Radius (RyR and SERCA)	a_{RyR}, a_{SERCA}	10 nm
# SERCA pumps in the SA head		36
# ions absorbed by one SERCA		2
# RyR in the SA base		36
# ions to activate one RyR		2
# calcium released from one RyR	n_{Ca}	varies (2-8)
# calcium released from one RyR	$\tau_{SER,TL}$	∞
