# Supplementary material for: Fast calcium transients in dendritic spines driven by extreme statistics
Source: PLoS Biol. 2019 Jun 4;17(6):e2006202. doi: 10.1371/journal.pbio.2006202 (PMC6548358; doi:10.1371/journal.pbio.2006202)
Supplement: S2 Table — (PDF) [file pbio.2006202.s011.pdf]

Table 2: Number of trials for numerical simulation results

Figure	Values
Fig 1E	16
Fig 2B	25
Fig 3C	25
Fig 3D N=500 & N=1000	750 & 1500
Fig 3E	10
Fig 3F	25
Fig 3G	10
Fig 3H	15
Fig 4A	20
Fig 4B	25
S1 Fig 4B	25
