# Supplementary material for: Fast calcium transients in dendritic spines driven by extreme statistics
Source: PLoS Biol. 2019 Jun 4;17(6):e2006202. doi: 10.1371/journal.pbio.2006202 (PMC6548358; doi:10.1371/journal.pbio.2006202)
Supplement: S1 Text — (PDF) [file pbio.2006202.s012.pdf]

S1 Text
Mathematical derivations of arrival times of the first and second ions

In this section, we derive the distribution of the first and second arrival time of the calcium ions released from the head and arriving to a single RyR. These expressions are then used in Fig 3D (main text) to compare the PDFs obtained analytically against Brownian simulation results.

During the Brownian motion inside a dendritic spine, calcium ions can be absorbed at the dendritic shaft, or they can return to the head after crossing into the neck any number of times. The pdf of no return can be computed by decomposing the total time (τ) as the sum of the time to reach the small window at the head-neck junction (τ^1) and the time spent in the spine neck (τ^2). The pdf of both times can be computed separately. According to the narrow escape theory [1], the distribution of arrival time of a Brownian particle at the entrance of the dendritic neck is Poissonian,

$$\Pr\{\tau^1 = s\} = \gamma e^{-\gamma s}, \tag{1}$$

where

$$\gamma^{-1} = \frac{|\Omega|}{4aD\left[1 + \dfrac{L(\mathbf{0}) + N(\mathbf{0})}{2\pi} a \log a + o(a \log a)\right]},$$

with $|\Omega|$ the volume of the spherical head, while a is the radius of the cylindrical neck [1] and $L(0)$ and $N(0)$ are the principal mean curvatures.

After the first particles reaches the cylinder (spine neck), we approximate its Brownian motion in the cylindrical domain by one-dimensional motion (1D). The pdf of arrival time of a Brownian particle to the end of an interval of length L is

$$\Pr\{\tau_2 = t - s\} = \sum_{n=0}^{\infty} (-1)^n \lambda_n e^{-D\lambda_n^2 (t-s)}, \tag{2}$$

where the eigenvalues are

$$\lambda_n = \frac{\pi}{L}\left(n + \frac{1}{2}\right). \tag{3}$$

We can now compute the pdf of the total time τ:

$$\begin{aligned}\Pr\{\tau_1+\tau_2=t\} &= \int_0^t \Pr\{\tau_2=t-s|\tau_1=s\}\Pr\{\tau_1=s\}ds \quad (4)\\ &= \gamma\int_0^t e^{-\gamma s}\sum_{n=0}^{\infty}(-1)^n\lambda_n e^{-D\lambda_n^2(t-s)}ds \quad (5)\\ &= \gamma\sum_{n=0}^{\infty}(-1)^n\left[\frac{e^{-D\lambda_n^2 t}-e^{-\gamma t}}{\gamma-D\lambda_n^2}\right]. \quad (6)\end{aligned}$$

This is the pdf of a Brownian particle's the arrival time at the base of a spine. This is a process with two timescales: one is dictated by diffusion and the other is Poissonian.
To compute the pdf of the shortest escape time τ^a with returns, that is the when a particle can return inside the head, we use Bayes' law for the escape density, conditioned on any number of returns, given by

$$\Pr\{\tau^a=t\}=\sum_{k=0}^{\infty}\Pr\{\tau^a=t|k\}\Pr\{k\}, \quad (7)$$

where $\Pr\{k\}=\frac{1}{2^k}$ is the probability that the particle returns k times to the head. The particle hits the stochastic separatrix [2] and then returns to the head, before reaching the dendrite. The probability of the escape, conditioned on k returns, $\Pr\{\tau^a=t|k\}$, can be computed from the successive arrivals times to the stochastic separatrix, $\tau_1,..\tau_k$, so that

$$\Pr\{\tau^a=t|k\}=\Pr\{\tau_1+..+\tau_k=t\}. \quad (8)$$

Assuming that the arrival time to the stochastic separatrix is Poissonian with rate λ_S [1], we obtain that

$$\Pr\{\tau_1+..+\tau_k=t\}=\lambda_S\int_0^t\frac{(\lambda_S s)^{n-1}}{(n-1)!}f(t-s)\,ds, \quad (9)$$

where $f(t)$ is the pdf of the time to escape the head entering the neck and returning to the head which we approximated by 4. Therefore,

$$\Pr\{\tau^a=t\}=\frac{1}{2}f(t)+\sum_{n=1}^{\infty}\int_0^t\lambda_S\frac{(\lambda_S s)^{n-1}}{(n-1)!}f(t-s)ds\frac{1}{2^k}. \quad (10)$$

Finally,

$$\Pr\{\tau^a=t\}=\frac{1}{2}f(t)+\int_0^t\exp(-\lambda_S s/2)f(t-s)ds, \quad (11)$$

Expression (4) with $\lambda_S = \gamma$ gives the final expression for the pdf of the escape time

$$
\begin{aligned}
f_{return}(t) &= \Pr\{\tau^a = t\} \qquad (12)\\
&= \frac{1}{2} f(t) + \gamma \sum_{n=0}^{\infty} (-1)^n \frac{\lambda_n \gamma^2}{4(\gamma - D\lambda_n^2)} \left[\frac{e^{-\gamma t/2} - e^{-\gamma t}}{\gamma/2} - \frac{e^{-\gamma/2t} - e^{-D\lambda_n^2 t}}{D\lambda_n^2 - \gamma} \right].
\end{aligned}
$$

The maximum of f_{return} is achieved at the point $t_{max} \approx \frac{2}{\gamma} \log 2$. The pdfs of the first and second arrivals are thus given by

$$
\begin{aligned}
f_{min}^{(1)}(t) &= \Pr\{\tau = min(t_1, \ldots, t_N) = t\}\\
&= N \left(1 - \int_0^t f_{return}(s) ds \right)^{N-1} f_{return}(t).
\end{aligned}
$$

In the Poissonian approximation, the pdf of the arrival time $\tau^{(2)}$ of the second fastest particle is given by

$$
f_{min}^{(2)}(t) = \Pr\{\tau^{(2)} = t\} = N \int_0^t f_{min}^{(1)}(t-s) f_{min}^{(1)}(s) ds. \qquad (13)
$$

The pdfs of the fastest and second fastest arrival times are computed from equation (12). We used expression 13 to compare this analytical result and the Brownian simulations in Fig 4B of the main text.

References

1. Holcman D, Schuss Z. Stochastic Narrow Escape in Molecular and Cellular Biology: Analysis and Applications. Biological and Medical Physics, Biomedical Engineering. Springer New York; 2015. Available from: https://books.google.fr/books?id=X0iGCgAAQBAJ.

2. Schuss Z. Theory and applications of stochastic processes: an analytical approach. vol. 170. Springer Science & Business Media; 2009.
